# Harnessing Nature for Breast Cancer Management: Effects of Fisetin-Loaded Nigellasomes Embedded in Microneedles Improve Tumor Suppression and Reduce Oxidative Stress

**DOI:** 10.3390/pharmaceutics17111392

**Published:** 2025-10-27

**Authors:** Sammar Fathy Elhabal, Eman Mohammed Ali, Sandra Hababeh, Fatma E. Hassan, Suzan Awad AbdelGhany Morsy, Dalia Ahmed Elbahy, Sahar K. Ali, Khaled M. Allam, Ibrahim Mousa, Marwa A. Fouad, Ahmed Mohsen Elsaid Hamdan

**Affiliations:** 1 Department of Pharmaceutics and Industrial Pharmacy, Faculty of Pharmacy, Modern University for Technology and Information (MTI), Mokattam, Cairo 11571, Egypt; 2 Department of Clinical Pharmacology, Faculty of Medicine, Sohag University, Sohag 82515, Egypt; 3 Department of Pharmaceutics, College of Pharmacy, King Saud University, Riyadh 11451, Saudi Arabia; 4 Medical Physiology Department, Faculty of Medicine, Cairo University, Kasr Alainy, Giza 11562, Egypt; 5 General Medicine Practice Program, Department of Physiology, Batterjee Medical College, Jeddah 21442, Saudi Arabia; 6 Department of Clinical Pharmacology, Faculty of Medicine, Alexandria University, Dr. Fahmi Abdelmeguid St., Mowassah Campus, Alexandria 21561, Egypt; 7 MBBS Program, Pathological Sciences Department, Fakeeh College for Medical Sciences, Jeddah 21461, Saudi Arabia; 8 Department of Clinical Pharmacology, Faculty of Medicine, Zagazig University, Zagazig 44519, Egypt; 9 Department of Pharmacognosy, Faculty of Pharmacy, Qena University, Qena 83523, Egypt; 10 Pharmaceutics Department, Faculty of Pharmacy, Sinai University, Al-Arish 16020, Egypt; 11 Department of Pharmaceutics and Pharmaceutical Technology, Faculty of Pharmacy, Deraya University, Minia 61768, Egypt; 12 Department of Pharmacy Practice, Faculty of Pharmacy, University of Tabuk, Tabuk 71491, Saudi Arabia

**Keywords:** natural products, fisetin, *Nigella sativa* oil, Ehrlich carcinoma, breast cancer, caspase-3, Gas Chromatography–Mass Spectrometry, nanovesicles, oxidative stress

## Abstract

**Background**: Natural compounds such as fisetin have promising in breast cancer treatment, but their poor pharmacokinetics limit their therapeutic application. This study utilized a synergistic approach by combining fisetin-loaded *Nigella sativa* (N.S.) oil nanovesicles (FIS-NSs) and carbohydrate-based microneedles (FIS-NSs-MNs) to improve breast cancer management. **Methods**: Chemical composition of NS petroleum ether extract using gas chromatography–mass spectrometry (GC/MS). FIS-NSs were prepared and characterized for particle size, polydispersity, zeta potential, encapsulation efficiency, and stability. These vesicles were embedded into gelatin, hyaluronic acid, and carboxymethyl cellulose microneedles. In vitro drug release, ex vivo permeation, cytotoxicity against breast cancer cells, and in vivo antitumor efficacy in Ehrlich tumor models were evaluated. **Results**: Optimized FIS-NSs displayed nanoscale size (190 ± 0.74 nm), low P.D.I (0.25 ± 0.07), high surface charge (+37 ± 0.57 mV), and high encapsulation (88 ± 0.77%). In vitro investigations showed sustained FIS release (~85% over 72 h), while ex vivo permeation showed higher absorption than free fisetin. Both FIS-NSs and FIS-NSs-MNs showed dose-dependent cytotoxicity against breast cancer cells, with lower IC_50_ than free fisetin (24.7 µM). In vivo, FIS-NSs-MNs and tumor burden inhibition (~77%), reduced oxidative stress (54%), restored antioxidant defenses, and decreased inflammatory markers. Immunohistochemical analysis for caspase-3 showed apoptosis activation within tumor tissues. **Conclusions**: These findings demonstrate that FIS administration via NS-MNs improves drug stability, penetration, and apoptotic activity, resulting in enhanced anticancer effects. This innovative nanovesicle–microneedle platform provides a non-invasive, effective, and patient-friendly approach for the effective treatment of breast cancer, with potential for broader applications in oncological nanomedicine.

## 1. Introduction

Breast cancer (BC) is the most common diagnosed cancer in women and the fifth leading cause of cancer death worldwide. Relative to lung cancer, breast cancer is now the most occurring in the world causing one out of every four cases of cancer in women [1,2]. Today, various protocols of treatment of BC have been adopted. The incidence and death rates are still significantly increased; the predicted rates of increase are 40% in 2040 compared to 2020, particularly in developing countries. Modern multimodal therapeutic approaches have emerged to manage breast cancer BC [3]. However, conventional systemic chemotherapeutic agents remain the mainstay of treatment at various stages, in spite of numerous problems that arise during their application [4,5]. The principal challenges for using conventional chemotherapeutic agents include severe side effects and resistance because of their lack of tissue selectivity and their quick clearance. Fortunately, herbal medicines offer new phytochemicals as effective treatments for a wide range of cancers. They provide the industry and health community with safe, effective, and low-cost alternative cytotoxic chemicals such as Taxol, Polyphenols, Terpenoids, and Alkaloids. Several in vitro and in vivo studies have shown that natural phytochemicals have significant anticancer activity against various cancers [6,7].

Flavonoids have recently received a lot of attention as anticancer agents due to their numerous biological activities, including antioxidant, antitumor, anti-inflammatory, immune-boosting, and hormonal modulating properties [8,9]. Flavonoids are plant polyphenols that have many evidence-based beneficial impacts for the treatment of breast cancer. They were shown to sequester cancer cell growth and division through targeting the cellular cycle subcellular targets, promoting necrosis and apoptosis of cancer cells [10]. Potent therapeutic properties and low systemic toxicity, compared to the synthetic drugs, paved the way for flavonoids to be used as monotherapy or an adjuvant to conventional chemotherapeutic agents, against various types of cancer aiming to improve patients’ response and quality of life [11].

Fisetin(3,3′,4′,7-tetrahydroxyflavone) is a polyphenolic flavonoid found in numerous fruits and vegetables, such as strawberries, apples, grapes, persimmons, onions, and cucumbers, with concentrations ranging from 2 to 160 μg/g [12,13]. It is present in numerous dietary supplements to improve health as an anti-inflammatory, antioxidant, and neuroprotective agents [14,15]. Fisetin (FIS) has been extensively studied as a potential anticancer agent for a variety of tumor types, including lung, prostate, oral, ovarian, pancreatic, liver, and colorectal cancer. It slows tumor growth, angiogenesis, invasion, and metastasis, it is also described as a multi-target entity capable of selectively modifying various molecular and signaling pathways in cancer cells while having no adverse effect on normal cells. Moreover, it circumvents the drug resistance commonly acquired by cancer cells in response to using standard chemotherapeutic agents due to its diverse cytotoxic effects [16]. FIS has numerous therapeutic benefits as an anticancer drug against various types of BC [17]. It has anticancer properties for both hormonal and non-hormonal types of breast cancer. Therefore, FIS may be an effective surrogate in the treatment of breast cancer. In spite of its promising in vitro anticancer activity against breast cancer, the in vivo applications are limited by its inadequate physicochemical properties, such as insufficient aqueous solubility (<1 mg/mL) and bioavailability. Consequently, numerous drug delivery systems (DDSs) have been formulated and explored in order to enhance the pharmaceutical properties of FIS and thereby increase its therapeutic efficacy [18,19].

*Nigella sativa* (N.S.), also known as black seed, black cumin, or black caraway, belongs to the Ranunculaceae family [20]. Black cumin seeds have a longstanding history of usage in traditional medicine due to their recognized therapeutic properties [21]. It comprises chemicals utilized as growth promoters and immune system enhancers for many aquatic species, which have been linked to significant anticancer activities [22]. Multiple studies have shown that N.S. extracts and their active components can suppress tumor growth in diverse cancer cell lines and animal models, cause apoptosis, and hinder cell migration and proliferation [23]. The plant also possesses significant anti-inflammatory and analgesic properties, indicating its potential utility in alleviating pain and inflammation. The principal saturated fatty acids in N.S. oil comprise linoleic acid (C18:2), oleic acid (C18:1), palmitoleic acid (C16:1), and eicosanoic acid [24,25]. Gebicki and Hicks discovered that unsaturated fatty acids could form closed lipid bilayer membrane vesicles termed ufasomes [26]. Nigellasomes represent a new class of bioactive nanovesicles derived from the unsaturated fatty acid content of *Nigella sativa* oil, integrating intrinsic therapeutic lipids within the vesicular bilayer to enhance stability, permeability, and biological synergy. Nigellasomes demonstrate superior dynamism compared to double-chain amphiphiles and micelles owing to their single-chain structure [27]. These possess numerous advantages compared to phospholipid vesicles, including enhanced stability, the capacity for fusion, improved cell membrane fluidity, and the capability to disrupt tight junctions. However, there is little research on unsaturated fatty acid vesicles for cancer treatment. Further research is necessary to fully understand [28] their biological effects and physicochemical properties, especially regarding transdermal distribution [29]. Transdermal dosage forms are being studied as a possible alternative to traditional drug delivery methods for the treatment of breast cancer, as they have several advantages, including minimal invasiveness, reduced systemic exposure, and avoidance of first-pass metabolism [30,31].

Microneedle array patches (MNs) have been identified as effective strategies for transdermal drug delivery [32]. Localized drug delivery methods in BC treatment have been examined for their painlessness and efficacy. Micron-scale needles breach the stratum corneum barrier, forming microchannels within the epidermis or upper dermis [33]. This technique facilitates direct drug delivery to subcutaneous tumor tissue. MN’s controlled drug release facilitates more drug accumulation in tumor tissues, reducing negative effects on other body tissues [34]. Dissolving microneedles (MNs) are composed of water-soluble, biocompatible, and biodegradable polymeric matrices that are biodegradable when skin and subcutaneous fluids release their cargo [35]. MNs may be able to improve the efficacy of breast cancer drugs, but there remain issues and limitations. The limited number of drugs that can be loaded may influence how well they can be used commercially. Using nanosized drug carriers in conjunction with an MN system can improve drug delivery by controlling release, targeting tumors, and allowing cells to absorb the drugs more easily. Bioactive nanosystems can also enhance the efficacy of antitumor drugs [36,37]. This work hypothesizes that fisetin-loaded Nigellasomes—bioactive nanovesicles generated from *Nigella sativa* oil embedded in dissolving microneedles—can boost drug penetration, stability, and anticancer activity in comparison to standard liposomal systems.

From this perspective, from this perspective, we investigated the incorporation of fisetin into *Nigella sativa* oil-based nanovesicles (‘Nigellasomes’) to enhance its delivery and anticancer efficacy; the approach is to load FIS into a predesigned nanoparticle formulation, which would be called “Nigellasomes”. These Nigellasomes–Fisetin nanovesicles will be formulated as bioactive vesicular nanocarriers using the 3^3^-factorial design. Designed Nigellasomes would be studied in vitro to investigate the effect of different formulation variables (*Nigella sativa* oil, cholesterol, and Span 60 concentrations) on Nigellasomes properties (including particle size (P.S), polydispersity index (P.D.I), zeta potential (Z.P) and entrapment efficiency percentage (E.E%). Afterwards, the selected Nigellasomes system was further optimized via incorporation into gelatin, hyaluronic acid, and CMC microneedles, to enhance penetration to breast tissues, reduce the elimination of the formulation from cancer tissues and enhance adhesion to the cancer surface.

## 2. Materials and Methods

### 2.1. Chemicals and Reagents

Fisetin and Span^®^ 60 were obtained from Sigma-Aldrich, Chemical Co., St. Louis, MO, USA. *Nigella sativa* seeds were procured from the Haraz Company, which is based in Cairo, Egypt. Gelatin (G6317, MACKLIN), hyaluronic acid (H823435, MACKLIN), and CMC (carboxymethyl cellulose) (#CC0112, Leagene, Beijing, China) were also acquired. All solvents (HPLC-grade), including ethanol, ethyl acetate, and petroleum ether (b.p. 60–80 °C), were obtained from Thermo Fisher Scientific (Pittsburgh, PA, USA).

### 2.2. Fixed Oil Extraction and Separation from Seeds of Nigella sativa

The seeds (45 g) were pulverized into a fine powder. Then, the oil was extracted using a Soxhlet apparatus (Buchi B-811, Buchi Labortechnik AG, Flawil, Switzerland) for approximately 6 h with ethanol until exhaustion. The crude ethanol extract (15 g) was suspended in distilled water and then fractionated with petroleum ether and ethyl acetate using the liquid–liquid technique. Each step’s organic phase was concentrated separately under vacuum, providing the petroleum ether fraction (6 g), which contained the seed oil, the ethyl acetate fraction (1 g), and the watery fraction (5 g). The oil fraction was stored at 4 ± 1 °C and analyzed using gas chromatography–mass spectrometry (GC/MS) [38].

### 2.3. GC/MS Analysis of the Isolated Nigella sativa Oil

The chemical composition of samples was evaluated using a trace GC-TSQ mass spectrometer (Thermo Scientific, Austin, TX, USA) and a direct capillary column TG-5MS (30 m × 0.25 mm × 0.25 µm film thickness). The oven temperature began at 50 °C and climbed at a rate of 50 °C per minute for 4 min, eventually reaching 250 °C. It was then heated to a final temperature of 300 °C for the next 2 min, increasing by 25 °C every minute. The MS transfer line and injector were maintained at 260 and 270 degrees Celsius, respectively. Helium was employed as the carrier gas, with a constant flow rate of 1 mL/min. After a 4-min solvent delay, diluted 1 µL samples were automatically injected into an Autosampler AS1300 coupled to a GC in split mode. In full scan mode, electron ionization (EI) mass spectra were recorded at 70 eV and ranged from 50 to 650 m/z. The ion source temperature of 200 °C was used. The components were identified by comparing their mass spectra with those from the NIST 14 and WILEY 09 mass spectral databases [39,40].

### 2.4. Fisetin High-Performance Liquid Chromatography Analysis

Fisetin (purity ≥ 98%) was analyzed using high-performance liquid chromatography (HPLC) with a reverse-phase C18 column (Phenomenex, Torrance, CA, USA) of 250 mm × 4.6 mm and 5 µm particle size. The mobile phase contained acetonitrile and 0.05 M sodium dihydrogen phosphate buffer (NaH_2_PO_4_) in a 25:75 (*v*/*v*) ratio. A UV detector calibrated to 358 nm, the wavelength of maximal absorption for fisetin, was used for detection, with a flow rate set to 1.0 mL/min. The approach was very linear, with a correlation coefficient (R^2^) of 0.999. During sample collection, 200 μL aliquots were extracted at predetermined intervals and immediately substituted with an equivalent volume of pre-warmed phosphate-buffered saline (PBS, pH 7.4) to sustain sink conditions and avoid external auto-concentration. The total amount of FIS emitted was measured over time using the standard calibration curve obtained from the known concentrations [41,42].

### 2.5. Animals

Female Swiss albino mice (5–7 weeks old, 20 ± 5 g) were obtained from the Animal House at the National Research Center in Giza, Egypt. They were acclimatized to a 12-h light/dark cycle and kept at a constant temperature of 22–24 °C for two weeks with free water and food. All animal experiments adhered to the National Institutes of Health’s Guide for the Care and Use of Laboratory Animals and the ARRIVE standards (National Experiments Council, 2011). The Experimental Protocol was authorized by Cairo University’s Faculty of Pharmacy’s Research Ethics Committee under the ethical approval number, 3770. At the end of the treatment, mice were anesthetized with a topical mixture of proparacaine hydrochloride (Alcaine^®^, Alcon Laboratories, Inc., Geneva, Switzerland) and benoxinate hydrochloride (BNP), and blood samples (0.5 mL) were collected from their retro-orbital plexus using heparinized capillaries and placed immediately on ice in EDTA-coated tubes. Samples were stored at −20 °C until they were analyzed. Mice starved overnight. The mice were subsequently sedated with xylazine (1 mg/kg) and ketamine (75–100 mg/kg) before being decapitated. Breast tumors were excised, numbered, weighed, and measured using a precision caliper with a 1 mm resolution. A portion of the breast tumor tissues removed from dead mice were rinsed with ice-cold saline to eliminate blood. To avoid protein degradation, each tissue sample was dried on sterile filter paper, weighed, and homogenized on ice in cold phosphate-buffered saline (PBS, pH 7.4) using a Teflon-glass homogenizer (IKA, Staufen, Germany). Homogenization was carried out at a ratio of 1 g tissue to 9 mL of buffer, yielding a 10% homogenate (*w*/*v*). The homogenates were centrifuged at 10,000× *g* for 15 min at 4 °C. The supernatants were carefully collected for biochemical analysis. Before analysis, homogenate aliquots were kept at −80 °C to preserve enzyme activity and prevent oxidation. The remaining half was processed for histopathological examinations.

### 2.6. Methods

#### 2.6.1. Design of Experiments Fisetin–Nigellasomes (FIS-NSs)

The FIS-NSs were tuned with a three-factor, three-level factorial design. Design-Expert^®^ program (version 13, Stat Ease Inc., Minneapolis, MN, USA) produced 15 FIS-NSs runs. The independent and dependent variables were determined by studying literature and conducting formulation studies. The independent variables were N.S. (X1), Span 60 (X2), and cholesterol (X3) concentrations in milligrams. The dependent responses comprised particle size (Y1), polydispersity index (Y2), zeta potential (Y3), and entrapment efficiency percentage (E.E%: Y4). ANOVA was used to determine statistical significance for each response. Table 1 shows the independent factors utilized for FIS-NSs optimization. Design-Expert^®^ software (Version 13, Stat-Ease Inc., Minneapolis, MN, USA) was used to create 3D response surface diagrams. The ideal NTD-FIS-NSs for simulation were found utilizing a numerical point prediction optimization method based on the desirability function. The best formulation was found by maximizing E.E% and Z.P (absolute value) while reducing P.S and P.D.I. The concentration ranges of N.S. oil, cholesterol, and Span 60 were calculated using preliminary screening studies that assessed vesicle size, dispersion stability, and fisetin solubility. Previous optimization studies on different nanovesicle systems for lipophilic flavonoids provided additional guidance for these quantities [9,27,43]. The chosen ranges ensured nanosized vesicle production with good encapsulation efficiency, positive surface charge, and adequate formulation stability, justifying their selection for factorial design optimization.

#### 2.6.2. Preparation and Optimization of Fisetin–Nigellasomes (FIS-NSs)

**Figure 1:**
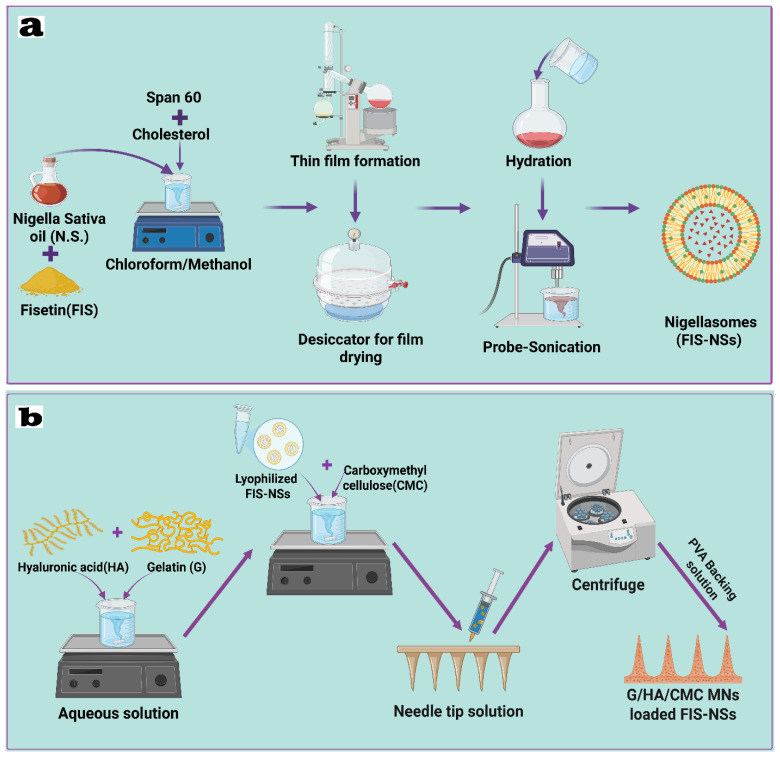
(**a**) Schematic representation of the Fisetin–Nigellasomes nanovesicles process. (**b**) Fabrication of Gelatin/Hyaluronic acid/Carboxymethyl cellulose microneedles (G/HA/CMC MNs) loading optimized Fisetin–Nigellasomes process.

The thin-film hydration process was used to create FIS-NSs with little modification. A round-bottom flask was used to dissolve the calculated amounts of FIS (6 mg), Span 60, cholesterol, and N.S. in a 1:110 mL methanol/chloroform solution. The Stuart rotary evaporator (RE300, Wolf Laboratories, North Yorkshire, UK) was used to evaporate the organic solvent was evaporated under vacuum at 60 °C and 100 rpm using a rotary evaporator (RE-300, Wolf Laboratories, UK) [44]. Evaporation was completed when a thin, dry, clear layer formed on the flask wall. To remove any organic solvent residues, the film was vacuum dried in a desiccator for two hours. To hydrate the film, add 10 milliliters of phosphate-buffered saline (PBS, pH 7.4). FIS-NSs were produced by hydrating at 60 °C for one hour at 100 rpm. A Sonix TV immersion sonicator (Model SS-series, North Charleston, SC, USA) was used for 10 min to shrink the vesicles. Figure 1a shows the customized nanosuspensions of FIS-NSs, which were kept at 4 °C overnight before further characterization [45].

#### 2.6.3. Physicochemical Characterization of Fisetin–Nigellasomes (FIS-NSs)

##### Particle Size, Polydispersity Index, and Zeta Potential

A small amount of Fisetin–Nigellasomes (FIS-NSs) formula was suspended in Milli-Q water and sonicated for 15 min. Then, part of each suspension was placed in the cuvette to estimate the P.S, P.D.I, and Z.P at 25 °C using the dynamic light scattering (DLS) Zetasizer equipment Nano ZS (Malvern Instruments, Worcestershire, UK) with a at 25 °C [46].

##### Entrapment Efficiency

The entrapment efficiency percentage of FIS-loaded Nigellasomes was indirectly estimated by determining the unentrapped drug amount in the supernatant after cooling centrifugation (13,000 rpm at 4 °C for 90 min). The supernatant was diluted appropriately, and the absorbance was spectrophotometrically measured at 360 nm using HPLC (Phenomenex, USA) as mentioned before [47]. The blank was the supernatant of plain Nigellasomes. The E.E% was calculated using Equation (1).

(1) $$\mathrm{EE}\;\%=\;\frac{\mathrm{FIS}\;\mathrm{Total}\;\mathrm{amount}-\mathrm{FIS}\;\mathrm{unentrapped}\;\mathrm{amount}}{\mathrm{FIS}\;\mathrm{Total}\;\mathrm{amount}}\times 100$$

#### 2.6.4. Evaluation of the Optimized Fisetin–Nigellasomes (FIS-NSs)

##### Transmission Electron Microscopy (TEM)

The optimized Fisetin–Nigellasomes (FIS-NSs) were morphologically examined using a TEM (JEM-100S microscope; JOEL Ltd., Tokyo, Japan). Briefly, 1 mL of the nanovesicle suspension was diluted tenfold with purified water. Samples were negatively stained with 2% (*w*/*v*) phosphotungstic acid (pH 7.0) for 1 min before imaging under TEM (JEM-100S, JEOL, Japan) at ×80,000 magnification. Images included a 500 nm scale bar. A drop of diluted suspension was applied to the surface of carbon-coated copper grids (200 mesh; Science Services; Munich, Germany). The excess substance was then removed using filter paper, leaving a thin coating that was spread over the holes and permitted to cure for 10 min at room temperature before examination [48]. The Digital Micrograph and Imaging Viewer applications were used for both image capture and analysis.

##### Fourier Transform Infrared Spectroscopy (FTIR)

The FTIR spectra of FIS, plain Nigellasomes, and optimized Fisetin–Nigellasomes (FIS-NSs) were detected via an FTIR Spectrophotometer (Madison Instruments, (Madison Instruments, Middleton, WI, USA)A hydrostatic press was adopted to prepare potassium bromide discs. The scanning range was from 500 to 4000 cm^−1^ [12,49].

##### Differential Scanning Calorimetry (DSC)

The DSC thermogram depicts the thermal characteristics using a Differential Scanning Calorimeter (DSC-60, Shimadzu Corporation, Kyoto, Japan) [standardized with indium (m.p = 156.6 °C, purity of 99.99%), heating rate = 10 °C/min] for FIS (4 mg). Plain Nigellasomes and optimized Fisetin–Nigellasomes (FIS-NSs) were heated in aluminium crimped pans at temperatures ranging from 30 to 450 °C with nitrogen gas flow (20 mL/min) [50,51].

##### Stability Study of the Optimized Fisetin–Nigellasomes (FIS-NSs)

The stability study for freshly prepared Fisetin–Nigellasomes (FIS-NSs) aqueous dispersions of the optimized formula was carried out under the International Council for Harmonisation (ICH) guidelines. FIS-NSs aqueous dispersions were stored in hermetically sealed glass bottles for 6 months at ambient and refrigerated temperatures (4 ± 1 °C) without agitation or stirring. The physical appearance, P.S, Z.P, P.D.I, E.E%, and drug retention (%) were assessed to evaluate their stability at zero time (preparation day) and following storage for 1, 2, 3, 4, 5, and 6 months [32,52]. Equation (2) was employed to calculate the percentage of drug retention.

(2) $$\mathrm{Drug}\;\mathrm{retention}\;(\%)=\frac{\mathrm{EE}\;\%\;\mathrm{at}\;\mathrm{each}\;\mathrm{time}\;\mathrm{interval}}{\mathrm{EE}\%\;\mathrm{interval}}\times 100$$

### 2.7. Fabrication of Carbohydrate-Based Hybrid Dissolving Microneedles (FIS-NSs-Loaded G/HA/CMC MNs)

The two-step micromolding process made carbohydrate-based hybrid dissolving microneedles (G/HA/CMC MNs). We mixed 5% (*w*/*v*) gelatin (G6317, MACKLIN) and 0.5% (*w*/*v*) hyaluronic acid (H823435, MACKLIN) in water and stirred it with a magnet at 40 °C until it was a smooth, bubble-free solution. Separately, the optimized (FIS-NSs) were freeze-dried (lyophilized) and then reconstituted in a 0.5% (*w*/*v*) carboxymethyl cellulose (CMC) solution (#CC0112, Leagene, Beijing, China) to improve viscosity and stability. The reconstituted FIS-NSs solution was gently mixed with the gelatin/HA blend at room temperature to create a uniform drug-loaded microneedle matrix [53,54]. Pipette 1 mL of the formulation into a pre-cleaned PDMS microneedle mold. Centrifuge at 4000 rpm for 15 min at 25 °C to fill the cavities and eliminate air bubbles. The filled molds were left to dry in a ventilated fume hood or drying oven at ambient conditions (25 °C) for 24 h to solidify the needle tips. After drying the primary drug-loaded matrix, 1 mL of a (3, 6, and 9%) (*w*/*v*) polyvinyl alcohol (PVA) solution (P875084, MACKLIN) was dissolved at 90 °C, cooled to room temperature, and was applied to the mold surface as the backing layer. The mold was left to dry for another 24 h under the same conditions to form a stable base supporting layer [32,55]. After solidifying, the microneedle patches were gently demolded by peeling away the PDMS mold, as shown in Table 2 and Figure 1b. They were then stored in a dedicator at room temperature (25 °C) until further testing or characterization.

### 2.8. Physical Characteristics of the Carbohydrate-Based Hybrid Dissolving Microneedles (G/HA/CMC MNs)

#### 2.8.1. Mechanical Strength and Penetration Capability Test

The TA-XT2 Texture Analyzer (CT3, Brookfield, Engineering, Middleboro, MA, USA) was utilized in compression mode to assess the compression properties of MN arrays. The MN array underwent compression against an aluminum block with a force of 30 N and a compression rate of 0.1 mm/s. The heights of MN arrays were assessed pre- and post-compression utilizing a stereomicroscope (C-B10+, Optika Microscope, Bergamo, Italy). The percentage decrease in MN height resulting from compression was determined using Equation (3). Parafilm M^®^ (Bemis Company Inc., Sheboygan Falls, WI, USA), a flexible olefin-based thermoplastic film that is often used to mimic the mechanical resistance of human skin, was used following a slightly modified procedure described by Elhabal [32]. To simulate manual insertion of MNs in the skin, an eight-layer film was folded, and MNs arrays were pressed against it with a 32 N force and 0.1 mm/s compression rate. The TA-XT2 Texture Analyzer was used for about 30 s. The array was then carefully removed from the Parafilm [56,57]. After unfolding the Parafilm layers, the holes created by insertion were examined using a C-B10 + stereomicroscope (Optika, Italy) by using Equation (4).

(3) $$\%\mathrm{Height}\;\mathrm{Reduction}=\frac{\mathrm{Initial}\;\mathrm{height}-\mathrm{Final}\;\mathrm{height}}{\mathrm{Initial}\;\mathrm{height}}\times 100\%$$

(4) $$\%\;\mathrm{Penetration}\;\mathrm{capability}=\frac{\mathrm{Number}\;\mathrm{of}\;\mathrm{holes}}{\mathrm{Total}\;\mathrm{needles}}\times 100\%$$

These parameters help determine the microneedles’ structural integrity under applied force and insertion efficacy into skin-like substrates.

#### 2.8.2. Determination of Drug Content

To determine the amount of drug incorporated into the MNs, Gelatin/Hyaluronic acid/Carboxymethyl cellulose microneedles loading freeze-dried optimized FIS-NSs-loaded G/HA/CMC MNs were dissolved for extraction. The MNs were put in distilled water with 2.5% (*v*/*v*) Tween 80, a surfactant that makes drugs easier to dissolve. At room temperature, the solution was stirred with a magnetic stirrer at 300 rpm for an hour to make sure the MNs matrix was completely dissolved. Then, methanol was added to the sample, and it was sonicated for five minutes to make sure that all of the Nigellasomes (NSs)-encapsulated FIS were released from carbohydrate-based hybrid dissolving microneedles. We filtered the solution and, using HPLC (Phenomenex, USA) as mentioned before, measured the amount of fisetin in the drug in µg or mg per microneedle array [58,59].

#### 2.8.3. Dissolution Time Assessment

To evaluate in vivo-like dissolution behavior, the MNs were applied, after euthanasia, full-thickness mouse skin was excised, hair was removed using an electric clipper, subcutaneous fat was thoroughly cleaned, and the skin was stored at −20 °C in phosphate-buffered saline until use (within one week). Manual thumb pressure facilitated the insertion of the MNs into the skin surface, after which a 5 g flat weight was applied to simulate prolonged attachment. Microneedle patches were systematically removed at specified time intervals of 2, 4, 6, and 8 min. The degree of needle tip dissolution was assessed using light microscopy to analyze the morphology of the extracted microneedles. This method facilitates the estimation of dissolution kinetics and the initiation rate of drug delivery, which is essential for time-controlled release applications [58,60].

#### 2.8.4. Water Loss During Drying (LOD) Test

The loss on drying (LOD) test was used to determine the residual moisture content in the microneedle matrix, which has a direct impact on storage stability and mechanical performance. Before molding, a 0.5 g sample of the MN formulation was accurately weighed and transferred into a clean mold. The sample was vacuum-dried in a dedicator at room temperature (25 °C) for 48 h [8,29]. After drying, the sample was reweighed, and the percentage of weight loss (i.e., moisture content) was calculated using the following Equation (5):

(5) $$\%\mathrm{LOD}\;=\;\frac{Initial\;weight-Final\;weight}{Initial\;weight}\times 100$$

A lower LOD (<10%) is generally preferred to ensure mechanical stability, prevent microbial growth, and increase shelf life.

#### 2.8.5. Characterization of Optimized Carbohydrate-Based Hybrid Dissolving Microneedles

##### Scanning Electron Microscopy (SEM)

A digital stereomicroscope (C-B10+, Optika Microscopes, Italy) was utilized to investigate the shape and dimensions of FIS-NSs-MNs arrays and tips. A scanning electron microscope (SEM) (model SM-IT200, manufactured by JEOL in Tokyo, Japan) was also utilized. The microneedle arrays were coated with gold through sputtering, and digital images were captured at a magnification of 150 times [51,61].

##### Fourier Transform Infrared (FTIR) Spectroscopy

FTIR spectra were obtained for plain carbohydrate-based hybrid dissolving microneedles and optimized Fisetin–Nigellasomes (FIS-NSs)-loaded carbohydrate-based hybrid dissolving microneedles (FIS-NSs-MNs) formula using the same method mentioned before.

##### Differential Scanning Calorimetry (DSC)

DSC were obtained for plain carbohydrate-based hybrid dissolving microneedles and optimized Fisetin–Nigellasomes (FIS-NSs)-loaded carbohydrate-based hybrid dissolving microneedles (FIS-NSs-MNs) formula using the same method mentioned before.

##### In Vitro Drug Release Study

FIS in vitro release studies from three formulations—the prepared optimized Fisetin–Nigellasomes (FIS-NSs), optimized Fisetin–Nigellasomes (FIS-NSs)-loaded carbohydrate-based hybrid dissolving microneedles (FIS-NSs-MNs), and pure FIS diffusion from aqueous dispersion—were examined in physiological pH phosphate buffer (PB, pH 7.4). The in vitro release investigation was conducted using vertical Franz diffusion cells with a surface area of 3.14 cm^2^ [62]. Sink conditions were maintained by incorporating 0.5% (*v*/*v*) Tween 80 in PBS (pH 7.4), in which FIS solubility (45 ± 2 µg/mL at 37 °C) exceeded by >20-fold the maximum cumulative FIS concentration in receptor fluid. The cumulative fisetin concentration remained below 10% of its saturation solubility throughout the experiment, ensuring valid release kinetics.

A cellulose membrane (cutoff 12,000–14,000 Da) was positioned between the receiver and the donor units [Spectrum Medical Industries Inc., Los Angeles, CA, USA]. This separates the diffusion cell’s donor compartment from the receptor compartment, and the membrane was equilibrated with the release medium solution overnight. Briefly, 4 mL of each formulation was placed in the donor compartment, and 50 mL of the release medium was deposited in the receptor compartment. The same procedure was performed for the aqueous medication solution in DW (S1), containing an equivalent number of FIS (4 mg). The diffusion cells were shaken in a thermostatically controlled incubator at 37 ± 0.5 °C and 100 rpm/min during the experiment (GFL Gesellschaft für Labortechnik, Burgwedel, Germany). A 3 mL sample was obtained from the receptor compartment at different time intervals, followed by resubstitution with an equivalent volume of fresh medium equilibrated at 37 ± 0.5 °C. The constructed aliquots were filtered through a 0.45 μm Millipore filter, and the released drug amount was evaluated spectrophotometrically at the predetermined λ_max_ = 360 nm using HPLC (Phenomenex, USA), as previously stated. Finally, the cumulative percent of FIS released was calculated and shown over time [63,64].

##### Ex Vivo Permeation Study and Kinetic Analysis

The penetration was studied for optimized FIS-NSs, FIS-NSs-MNs, and FIS solution in sheep skin tissue collected from an abattoir in Cairo, Egypt. Sheep skin was chosen because it has a similar lipid composition, hair follicle density, and dermal thickness to human skin, making it an acceptable surrogate for ex vivo transdermal research [65]. The skin penetration of several FIS formulations was measured ex vivo using a modified Franz diffusion cell equipment to determine the amount of FIS in the skin. A skin FIS solution like the drug, FIS-NSs, was applied to the sheep’s skin surface in the diffusion cell, along with FIS-NSs-MNs. Water at 37 °C ran through the water jacket around the receptor cell, and a Teflon-coated magnetic stir bar at the cell’s bottom maintained that the receptor volume was consistent [66,67]. Fisetin recovery from donors, receptor, and skin extracts yielded 96 ± 3%, showing minimal degradation. Skin integrity was verified using TEWL < 10 g m^−2^ h^−1^ and resistance > 20 kΩ cm^2^ before and after penetration. The diffusion medium consisted of phosphate buffer with a pH of 7.4. A sample of receptor media was obtained at specific time intervals (1, 2, 4, 6, 8, 24, 48, 72, and 80 h), and the concentration of FIS was determined. After 80 h, the skin was removed from the cell and homogenized with 5 mL of DMSO for 5 min. Kinetic analysis was investigating reaction mechanisms and quantitative modeling of reaction kinetics [68,69]. To gain insight into the FIS release mechanism from optimized FIS-NSs, FIS-NSs-MNs, and FIS solution formula, in vitro release outcomes were evaluated using various kinetic models, including zero and first orders, the Higuchi diffusion equation, and the Korsmeyer–Peppas semi-empirical model for the initial 60% release data, as outlined in Equation (6), to validate the mechanism.

Mt/M∞ = kt ^n^ (6)

In this context, Mt/M∞ represents the fraction of the drug released at time t, k denotes the release rate constant, and n indicates the characteristic diffusional exponent. Furthermore, an experimental model that appears to characterise the diverse release patterns exhibited by optimized FIS-NSs, FIS-NSs-MNs and FIS solution Weibull model (an empirical model) was utilised for the release data as outlined in Equation (7):

(ln [−ln (1 − F)] = β ln td + β ln t) (7)

In this context, F represents the fraction of the drug released at time t, while td indicates the lag period prior to the commencement of drug release. The exponent β characterizes the form of the release curve and indicates the drug transport mechanism within the nanoparticle matrix. The parameter β ≤ 0.75 indicates Fickian diffusion, whereas 0.75 < β < 1 represents a hybrid mechanism combining Fickian diffusion and Case II transport. A β value exceeding 1 indicates that the drug transport follows a complex release process. The model exhibiting the highest coefficient of determination (R^2^) is considered the most superior model [70,71].

### 2.9. In Vitro Cell Culture Studies

#### 2.9.1. Cell Line

The human cancer cell line MDA-MB-231 (ATCC HTB-26™) was procured from the American Type Culture Collection (ATCC) in Manassas, VA, USA, via VACSERA, Egypt, for in vitro cytotoxicity evaluations to test the effects of these formulations on the skin. Cells were grown in high glucose Dulbecco’s modified Eagle’s medium (DMEM, Sigma-Aldrich Co.), supplemented with 10% *v*/*v* fetal bovine serum (FBS) and 1% antibiotics (100 μg/mL streptomycin and 100 U/mL penicillin), at 37 °C in a 5% CO_2_ incubator (Binder, Tuttlingen, Germany) [72,73].

#### 2.9.2. Cellular Cytotoxicity

The cellular cytotoxicities of FIS and FIS-NSs were assessed utilizing the MTT test. Doxorubicin functioned as a positive control. MDA-MB-231 cells were plated in 96-well plates at a density of 5 × 10^3^ cells per well and incubated at 37 °C with 5% CO_2_ for 24 and 48 h. The medium was then substituted with MTT solution (0.5 mg/mL) and incubated for 3 h. The formazan blue crystals were solubilized in DMSO, and absorbance was quantified at 570 nm utilizing a Tecan Infinite F50 ELISA plate reader (Männedorf, Switzerland). Optical density was evaluated in comparison to the control, which signifies 100% cell viability. Cell viability percentages and IC_50_ values were ascertained [74,75].

### 2.10. In Vivo Study

#### 2.10.1. Induction of Breast Cancer in Mice

**Figure 2:**
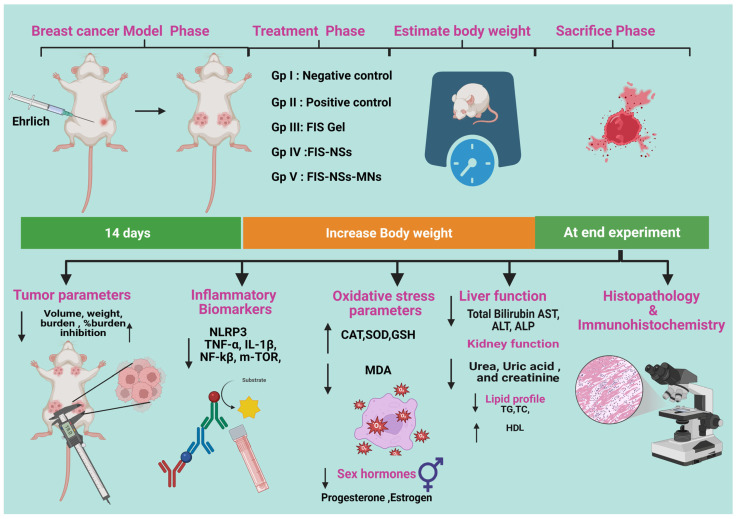
Schematic representation of the in vivo study utilizing breast cancer using Ehrlich model.

Female Swiss albino mice were selected according to the specifications of the Solid Ehrlich Carcinoma (SEC) model. The Ehrlich Ascites Carcinoma (EAC) cell line (ATCC CCL-77) was generously provided by the Pharmacology and Experimental Oncology Unit of the National Cancer Institute at Cairo University in Egypt. Each mouse received 0.2 mL of a 2 × 10^6^ cells/mL suspension into the left mammary fat pad. Tumor growth was monitored every 48 h with a digital caliper until volumes reached ≈100 mm^3^. All procedures were approved by the Faculty of Pharmacy Research Ethics Committee (Cairo University; approval No. 3770) (14 days post-inoculation) [76,77]. The mouse was euthanized, and cells were harvested from ascitic fluid using a syringe and diluted with normal saline (1:1). Viable cells were subsequently given subcutaneously (0.2 mL/mouse) into the left side of the recipient of the mammary fat pad of the mice. After 14 days, mice with SEC exhibited tumor mass development [78,79]. Fifty mice were randomly assigned to five groups (n = 10 each): Group I (healthy negative control group; healthy mice injected with saline), Group II (SEC model group, untreated), Group III (SEC treated with FIS incorporated in gel containing 2% (*w*/*w*) HPMC), Group IV (SEC treated with FIS-NSs incorporated in gel containing 2% (*w*/*w*) HPMC), and Group V (SEC treated with FIS-NSs incorporated in G/HA/CMC MNs). All different formulations were equivalent to 30 mg FIS/kg. Each MN array contained 92.4 ± 3.1 µg, eight arrays were applied per animal to provide roughly 30 mg FIS/kg. The arrays were replaced every other day for 14 days [80]. All treatments commenced on day 14 post-tumor induction and were administered transdermally every other day for a duration of two weeks, as seen in Figure 2.

#### 2.10.2. Estimation of Skin Morphology and Body Weight

To assess morphological alterations in the skin overlaying and surrounding subcutaneous tumors, including epidermal/dermal architecture, ulceration, inflammation, vascular/lymphatic remodeling, fibrosis, and tumor–skin interface characteristics. Body weights were estimated for all the groups of animals just before the study and at the end of 4 weeks using an electrical balance (Sartorius BL210S, Sartorius AG, Göttingen) [81].

#### 2.10.3. Estimation of Tumor Parameters

The Vernier caliper was used to measure the tumor size and quantify it using the following formula [82]. To calculate tumor volume (cm^3^), use Equation (8):

Tumor volume = length × (width)^2^ × 0.5 (8)

The tumor load [83] was determined in the following Equation (9):

Tumor burden = ∑ tumor mass in a group (9)

The tumor burden reduction was determined in the following Equation (10):

(10) $$\%\;\mathrm{Tumor}\;\mathrm{burden}\;\mathrm{inhibition}=\frac{Tumor\;burden\;in\;Ehrlich\;tumor-burden\;in\;the\;test\;group}{Tumor\;burden\;in\;Ehrlich\;tumor}\times 100$$

The tumor weight was measured by electric balance and expressed in gram unit.

#### 2.10.4. Assessment of Inflammatory Biomarkers

To assess the anti-inflammatory and anti-proliferative effects of fisetin treatment in breast cancer, essential tissue biomarkers were evaluated using ELISA kits in accordance with the manufacturer’s guidelines (MyBioSource, San Diego, CA, USA). The chosen biomarkers were selected due to their mechanistic relevance to the established biological effects of fisetin, specifically its influence on inflammatory, proliferative, and angiogenic pathways. Examine the serum for NLRP3 (NOD-, LRR-, and Pyrin Receptor Protein 3), TNF-α (Tumor Necrosis Factor-Alpha), IL-1β (Interleukin-1 Beta), NF-κB (Nuclear Factor Kappa B, nuclear translocation), and mTOR (Mammalian Target of Rapamycin) [8,84], as depicted in Figure 2.

#### 2.10.5. Assay for Oxidative Stress Parameters

Oxidative stress was evaluated through both oxidant and antioxidant (enzymatic and non-enzymatic) parameters, including MAK085—malondialdehyde (MDA), MAK531—catalase (CAT), CS0009—superoxide dismutase (SOD) activity, and MAK517—reduced glutathione (GSH) (Sigma-Aldrich, St. Louis, MO, USA).

##### Assessment of Oxidant Biomarkers

The level of malondialdehyde (MDA), a marker of lipid peroxidation, in tissues was measured as described in [85,86]. This approach relies on the interaction of MDA with N-methyl-2-phenylindole to form a blue chromophore with a maximum absorbance of 586 nm. To start, combine 200 μL of mammary homogenate with 650 μL of 10 mM N-methyl-2-phenylindole in a 3:1 (*v*/*v*) acetonitrile/methanol solution. Next, 150 μL of 37% HCl was added to start the reaction. The mixture was incubated at 45 °C for one hour before measuring absorbance at 586 nm. The results were presented as nmol/mg protein [10,87].

##### Enzymatic Antioxidant Parameters

Catalase (CAT) activity in tissues was measured by monitoring the exponential breakdown of hydrogen peroxide (H_2_O_2_) at 240 nm [88,89]. The results were represented as (units/mg of protein). The mammary tissues were homogenized in PBS (pH 7.4, 8% *w*/*v*) and centrifuged at 15,000× *g* for 20 min at 4 °C. The absorbance was measured at 520 nm. Superoxide Dismutase (SOD) activity was determined using Nandi and Chatterjee’s technique, which is based on the enzyme’s ability to block the auto-oxidation of pyrogallol at alkaline pH. In brief, the reaction was carried out in 2 mL of 50 mM Tris-Cacodylate buffer (pH 8.5). To start the reaction, add 100 μL of newly produced 6 mM pyrogallol in 10 mM HCl to a buffer with 20 μL of mammary homogenate. The absorbance was measured at 440 nm for two minutes. SOD activity was measured in (units/mg of protein,) with one unit being the enzyme quantity required to block 50% of pyrogallol auto-oxidation [10].

##### Non-Enzymatic Antioxidant Parameters

Levels of reduced glutathione (GSH) were assessed using the method: one milliliter of the sample was treated with 1 mL of 5% TCA and subjected to centrifugation at 1200× *g* for 20 min. Subsequently, 0.5 mL of the supernatant was combined with 2 mL of 5,5′-Dithiobis (2-nitrobenzoic acid) (DTNB). The yellow complex formed was measured at 412 nm, with results expressed as nmol/mg protein [90,91].

#### 2.10.6. Liver Function Tests (LFTs), Kidney Function Tests and Sex Hormone Assessment

Renal functions (urea, uric acid, creatinine), total bilirubin, and the activity of aspartate aminotransferase were estimated. The activities of alanine transaminase and alkaline phosphatase, and estrogen and progesterone, were assessed using commercial reagent kits and a spectrophotometer.

#### 2.10.7. Lipid Profile

Triglyceride (TG) levels in female mouse plasma were determined using Rice’s (1970) method [92], total cholesterol (TC) levels were measured using Burstein (1970) method [93], high-density lipoproteins (HDL) were estimated using Burstein et al.’s (1989) protocol [94].

### 2.11. Histopathological Analysis

Breast tissues from decapitated rats were removed and preserved in a 10% formaldehyde solution. After 48 h, samples were dried with ethanol. Breast tissues were then fixed in paraffin and sliced into 5 µm sections by microtome. Hematoxylin/Eosin was used to stain the tissue, and the produced slides were examined under a light microscope [95].

### 2.12. Immunohistochemical (IHC) Examination of Caspase-3

Breast tissue samples were processed for immunohistochemical evaluation per the manufacturer’s instructions using an IHC staining kit (Invitrogen^TM^, Carlsbad, CA, USA). ABclonal’s caspase-3 primary antibody (Catalogue No. A11953) was used at a 1:100 dilution, and each group was incubated overnight per the antibody’s instructions. The percentage of reaction area in seven microscopic areas was assessed using ImageJ 1.53t, which was developed by Wayne Rasband and colleagues from the National Institutes of Health in the United States. Five non-overlapping fields were randomly picked at 40× magnification [96,97].

### 2.13. Statistical Analysis

Differences between groups were evaluated by one-way ANOVA followed by Tukey’s post-hoc test using GraphPad Prism 8.0 (*p* < 0.05 considered significant). Tumor volume, tumor weight, and biochemical parameters were all examined using one-way ANOVA followed by Tukey’s multiple-comparison test. Two-way repeated measures ANOVA was used to compare the progression of body weight and tumor volume over time. Results from both in vitro, ex vivo and in vivo are presented as mean ± SD (n = 10). The following notation was used: ns = not significant (*p* > 0.05), * (*p* < 0.05), ** (*p* < 0.01), *** (*p* < 0.001), and **** (*p* < 0.0001).

## 3. Results and Discussion

### 3.1. GC/MS Analysis of the Petroleum Ether Extract of Nigella sativa Seeds

**Figure 3:**
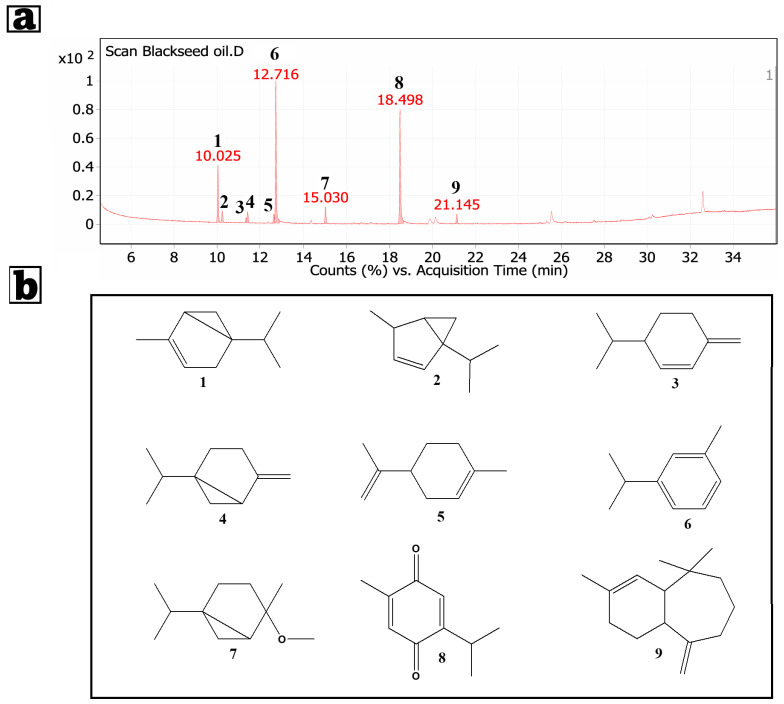
(**a**) GC/MS of the petroleum ether extract of *Nigella sativa* seeds. (**b**) Structure of the compounds, identified from GC/MS analysis of the petroleum ether extract of *Nigella sativa* seeds.

In the current study, the petroleum ether extract of *Nigella sativa* seeds was subjected to the GC/MS technique to identify its constituents. By GC/MS analysis of *N. Sativa* petroleum extract, nine compounds were identified (Figure 3). Relative area percentages and retention times of the identified compounds are listed in Table 3. GC/MS analysis of the *Nigella sativa* oil petroleum ether extract revealed a complex spectrum of bioactive compounds: p-cymene (Benzene, 1-methyl-3-(1-methylethyl)-, 37.14%), thymoquinone accounting for 32.36%, alpha-thujene (14.12%), cis-4-methoxy thujane (4.3%), and several additional monoterpenes. Some modest peaks may correspond to transient tautomeric or rearrangement species produced during electron-impact ionization; they have been identified as analytical artifacts rather than permanent metabolites [98]. Some modest peaks may correspond to transient tautomeric or rearrangement species produced during electron-impact ionization; they have been identified as analytical artifacts rather than permanent metabolites. Thymoquinone’s dominance is noteworthy for its potent anticancer capabilities, particularly in breast cancer models. Thymoquinone effects include cancer cell death, inhibition of proliferation and metastasis, and suppression of signaling pathways like NF-κB and Akt/mTOR, widely linked to breast cancer growth. Thymoquinone increases tumor cell susceptibility to traditional chemotherapeutic drugs, resulting in lower dosages, adverse effects, and increased safety. The common chemical p-Cymene has anti-inflammatory and antioxidant properties. When combined with thymoquinone and other oil terpenes, p-Cymene increases anticancer activity. This combination may enhance *Nigella sativa* oil’s anticancer properties by reducing oxidative stress in the tumor microenvironment and inhibiting inflammatory pathways that promote tumor growth and survival. Alpha-thujene, beta-phellandrene, and D-limonene monoterpenes enhance the oil’s therapeutic qualities. D-limonene inhibits the cell cycle and induces apoptosis in breast cancer cells, according to studies. Because of their antioxidant and anti-inflammatory properties, cis-4-methoxy thujane and similar compounds may protect normal tissues while also reducing anticancer therapy side effects [99]. Numerous GC/MS studies have revealed that the percentage composition of *Nigella sativa* (black seed) petroleum ether extract changes according to the seed’s geographic origin, extraction process, and whether the essential or fixed oil is studied. According to scientific literature, the essential oil of *Nigella sativa* is normally produced from seeds using extraction with petroleum ether, with thymoquinone (TQ; 2-methyl-5-(propan-2-yl)cyclohexa-2,5-diene-1,4-dione) accounting for up to 50% of the primary bioactive ingredients [100,101]. In one investigation, TQ concentrations were measured in 19 commercial oil formulations. TQ was also put into two serum samples to assess its recovery after deproteinization with toluene. Furthermore, one healthy volunteer swallowed 1 g and another 3 g of a highly concentrated *Nigella sativa* oil, and serum TQ levels were evaluated at 30 and 60 min after delivery [102]. Oleic acid and linoleic acid were found to be primary unsaturated fatty acids in our GC/MS investigation of *Nigella sativa* oil extract reported by Abo-Neima [103]. Hossain et al. found that these chemicals affect cardiac glycoside interactions with the Na^+^/K^+^-ATPase (sodium pump), which may contribute to the oil’s cardioprotective and bioactive characteristics [104].

### 3.2. Statistical Analysis of the Factorial Experimental Design Fisetin–Nigellasomes (FIS-NSs)

A factorial design was utilized to systematically study and optimize the effects of *Nigella sativa* oil concentration (X1), cholesterol concentration (X2), and Span 60 concentration (X3) on key responses for FIS-NSs; particle size (P.S), polydispersity index (P.D.I), zeta potential (Z.P.), and entrapment efficiency (E.E%) as shown in Table 1. The design enabled efficient screening of factor interactions and quadratic effects with a minimal number of experimental runs as shown in Table 4. Adequate precision for all responses exceeded 4, indicating a suitable signal-to-noise ratio and reliable model discrimination. The range of R^2^ values was 0.59 (for P.S) to 0.99 (for E.E%), showing good to excellent model fit, especially for E.E% (R^2^ = 0.9957, Adjusted R^2^ = 0.9879). The F-values for all responses were significant (*p* < 0.05), suggesting that the models effectively described the relationships between the variables and the responses. A strong correlation between predicted R^2^ and adjusted R^2^ was observed, further validating the models, particularly for E.E%, as indicated in Table 5.

### 3.3. Evaluation of FIS-NSs Formulations

#### 3.3.1. Particle Size, Polydispersity Index, and Zeta Potential

##### Particle Size (P.S)

**Figure 4:**
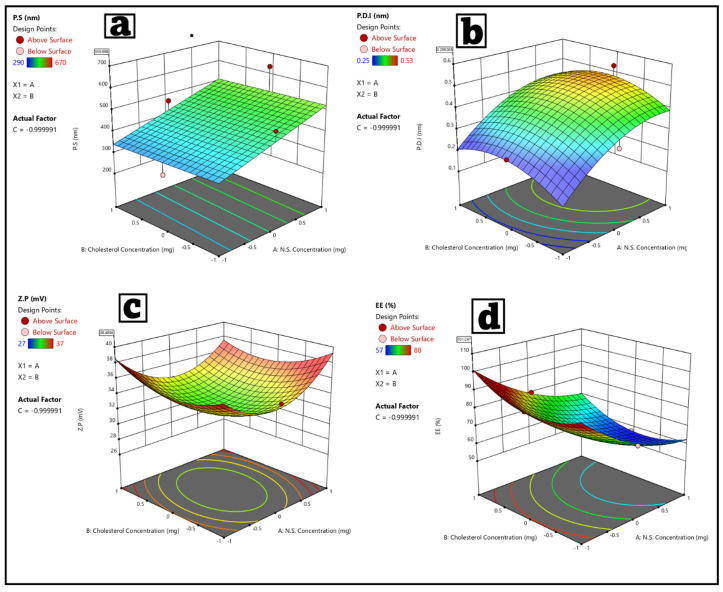
(**a**–**d**) 3D Response surface plot showing the effect of N.S. Conc (X1), Cholesterol Conc (X2), and Span 60 Conc (X3) on (**a**) particle size (P.S), (**b**) polydispersity index (P.D.I), (**c**) zeta potential (Z.P), and (**d**) entrapment efficiency percentage (E.E%).

The particle size of FIS-NSs varied from 190 ± 0.74 nm (minimum, Run 11) to 670 ± 0.76 nm (maximum, Run 9) among the formulations. Reducing particle size is essential for improved cellular uptake and transdermal delivery. The main factors identified were N.S. concentration (X1) and Span 60 concentration (X3), which yielded a *p*-value of 0.0078. The increase in Span 60 often resulted in the generation of smaller vesicles, which can be linked to improved surfactant coverage that stabilizes the interface and prevents aggregation. In contrast, the decrease in oil concentration in N.S. resulted in smaller vesicle sizes, which can be linked to a reduction in core volume and an improvement in packing efficiency [32,105]. The influence of cholesterol was minimal, aligning with its established role as a membrane stabilizer rather than a major determinant of particle size. The findings correspond with the recent study conducted by [31], which demonstrated that higher surfactant ratios resulted in a decrease in vesicle size in both niosomal and bilosomal systems [106]. The updated formulation (Run 11) achieved a particle size of 190 ± 0.74 nm, suggesting its applicability in drug delivery systems as shown in Figure 4a. Nanosized vesicles are more effective at bypassing biological barriers [8].

##### Polydispersity Index (P.D.I)

PDI values, indicating size distribution uniformity, ranged from 0.25 ± 0.07 (most monodisperse, Run 11) to 0.53 ± 0.07 (most polydisperse). Significant factors are N.S. concentration (X1) and cholesterol concentration (X2) (*p* = 0.0084). Lower N.S. concentration and higher cholesterol improved homogeneity, producing more uniform vesicle populations. Higher Span 60 also contributed to a lower P.D.I, in line with its role in stabilizing vesicle formation. The design revealed significant effects from both N.S. (X1) and cholesterol (X2) (*p* = 0.0084) as shown in Figure 4b. The adjusted formulation (Run 11) had a very good P.D.I, which improved product stability and batch-to-batch uniformity.

##### Zeta Potential (Z.P)

The formulations’ zeta potential (Z.P) values were all positive, with a range of +27 ± 0.83 mV to +37 ± 0.57 mV. This implies that the vesicles possess a net positive surface charge; this positive charge electrostatically repels particles, preventing them from aggregating or clustering. Consequently, the formulations are endowed with electrostatic stability, which is crucial for the preservation of colloidal dispersion over time. The concentrations of N.S. (factor X1) and Span 60 (factor X3) had a significant impact on Z.P values among the variables studied (*p* = 0.0074), suggesting that changes in these factors can greatly affect the surface charge of the vesicles. This trend could stem from the capacity of these components to enhance surface activity, thereby improving charge distribution on the vesicle surface and aiding in charge stability. Particles are unable to interact due to the strong repulsive forces acting between them, with zeta potential levels exceeding +30 mV generally considered a sign of high colloidal stability. The Z.P values obtained, particularly at the higher end of the spectrum, indicate that the formulations exhibit stability and are suitable for potential pharmacological or biological applications. The zeta potential serves as a crucial indicator of vesicle stability. Our results indicate that deliberate adjustments to formulation parameters, including N.S. and Span 60 concentrations, can enhance colloidal performance and optimize surface charge, as shown in Figure 4c.

#### 3.3.2. Entrapment Efficiency (E.E%)

EE% is a critical parameter reflecting the capacity of the formulation to encapsulate fisetin. Observed values ranged widely from 57 ± 0.75% (lowest, Run 12) to 88 ± 0.77% (highest, Run 11). Significant factors are all three of the independent variables; N.S. concentration (X1), cholesterol concentration (X2), and Span 60 concentration (X3) were significant (*p* < 0.0001). Elevated Span 60 and moderate cholesterol levels promoted higher E.E%, likely attributable to improved bilayer flexibility and optimum vesicle integrity. The optimal concentration of N.S. is crucial; inadequate levels reduce encapsulation, whereas excessive amounts may destabilize the vesicles. The optimized formula (Run 11) exhibited the highest encapsulation efficiency (E.E%) of 88 ± 0.77%, the lowest particle size (P.S) of 190 ± 0.74 nm, a low polydispersity index (P.D.I) of 0.25 ± 0.07, and a high zeta potential (Z.P.) of 37 ± 0.57 mV, as shown in Figure 4d, indicating its suitability for effective fisetin delivery.

#### 3.3.3. Optimization of FIS-NSs Formulations

Based on the desirability criterion, Design-Expert^®^software (Version 13, Stat-Ease Inc., Minneapolis, MN, USA) was utilized to obtain the optimum values of the independent variables. Optimized formulation (Run 11) successfully met all the design goals: minimal particle size and P.D.I and maximal zeta potential and entrapment efficiency. Factorial design efficiently elucidated the main and interaction effects of formulation variables, guiding selection of ideal conditions for FIS-NSs preparation. The statistical and experimental evaluations demonstrated the ability to adjust FIS-NSs characteristics through variations in N.S., cholesterol, and Span 60 concentrations. Table 5 illustrates a strong correlation between expected and observed values. The improved formulation represents a potential nanosystem for the effective encapsulation and distribution of fisetin, supported by strong experimental and model-based data.

### 3.4. Evaluation of the Optimized Fisetin–Nigellasomes (FIS-NSs)

#### 3.4.1. Transmission Electron Microscopy (TEM)

**Figure 5:**
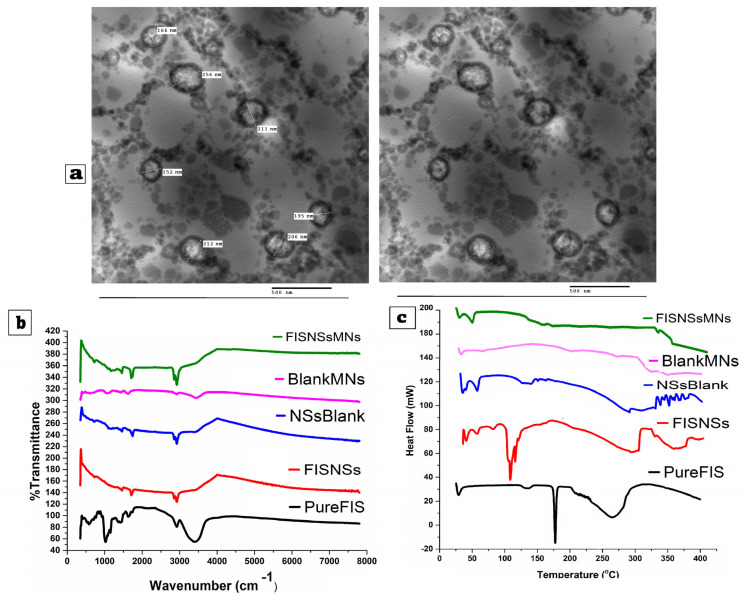
(**a**) TEM micrograph of optimized Fisetin–Nigellasomes (FIS-NSs) (Run11). Fourier Transform Infrared Spectroscopy (**b**) and Differential Scanning Calorimetry (**c**) for pure fisetin (Pure-FIS), fisetin-loaded nanosystems (FIS-NSs), blank nanosystems (NSs-Blank), and FIS-NSs-loaded G/HA/CMC MNs.

Transmission Electron Microscopy (TEM), employed to examine the morphology of the synthesized FIS-NSs, as shown in Figure 5a, indicated that the majority of the nanovesicles exhibit a spherical morphology with smooth, well-defined edges. The vesicles exhibited clear separation, with no signs of aggregation or fusion, indicating successful stabilization of the system. The TEM image indicated that the vesicles varied in size from 150 to 256 nm, with a mean size of approximately 190 nm. The TEM-derived mean diameter (~190 nm) was slightly smaller than the DLS size (~220 nm), which was expected due to drying shrinkage during sample preparation. Hence, vesicles were described as spherical and well-dispersed rather than absolutely “unilamellar”.

The values exhibit a strong correlation with the particle size distribution derived from dynamic light scattering (DLS) or Zetasizer analysis. This suggests that the formulation process is reliable and can be consistently reproduced. The monodispersity and non-aggregated characteristics of the vesicles can be attributed to the formulation’s elevated absolute zeta potential values. These values facilitate particle electrostatic repulsion, reducing agglomeration and stabilizing colloids. Encapsulation, cellular uptake, and release are enhanced in spherical and unilamellar vesicles for pharmaceutical administration. The nanosystem’s consistent size and shape distribution, as observed in TEM, suggests that consistent manufacturing improves therapeutic efficacy and batch uniformity [9,30].

#### 3.4.2. Fourier Transform Infrared Spectroscopy (FTIR)

The chemical structure and interactions of fisetin were analyzed using Fourier Transform Infrared (FTIR) Spectroscopy across several formulations, as shown in Figure 5b, for pure fisetin (Pure-FIS) and fisetin-loaded nanosystems (FIS-NSs). FTIR is an efficient tool for identifying functional groups and assessing potential interactions between pharmaceutical drugs and excipients through distinctive absorption peaks. The spectrum of pure fisetin displays distinct peaks that align with its functional categories. The significant absorption band observed at 3300 cm^−1^ is associated with the stretching vibrations of O-H bonds from phenolic hydroxyl groups. The peaks around 1650 cm^−1^ indicate flavonoid core C=O stretching, while peaks between 1500 and 1600 cm^−1^ indicate aromatic C=C stretching vibrations. Additional bands in 1000–1300 cm^−1^ suggest C-O stretching. These spectral features confirm the structural integrity and purity of the fisetin compound. In contrast, the FIS-NSs spectrum shows a noticeable decrease in the intensity of the characteristic fisetin peaks and slight shifts in their positions. Fisetin appears to be enclosed in the nanosystem and interacts with the carrier matrix via hydrogen bonding or van der Waals. The broadening of the O–H and C=O peaks support these interactions. This indicates that fisetin has infiltrated the nanoparticle system. The results have been used to verify the structural integrity and compatibility of the delivery strategies [17].

#### 3.4.3. Differential Scanning Calorimetry (DSC)

Differential Scanning Calorimetry is commonly utilized to assess the crystalline state of pharmaceuticals. Figure 5c displays the DSC thermograms for pure fisetin (Pure-FIS), fisetin-loaded nanosystems (FIS-NSs), and blank nanosystems (NSs-Blank). Pure fisetin (Pure-FIS) exhibited a distinct endothermic peak (melting point) in the range of approximately 134–138 °C, as indicated by the thermograms. The distinct peak observed indicates that FIS is crystalline, confirming its presence in a pure, untreated, crystalline state. The thermogram of the FIS-NSs indicates a significant disappearance or broadening of the distinct FIS melting endotherm observed in Pure FIS, accompanied by a broad endothermic event occurring at 115–120 °C. The absence of a distinct melting peak within the range of 134–138 °C indicates that fisetin has likely transitioned from its crystalline form to an amorphous or molecularly dispersed state within the nanosystem. The appearance of novel, less pronounced transitions. The thermograms show that (Pure FIS) presents a distinct acute endothermic peak, which aligns with a melting point of around 134–138 °C. The notable peak detected validates the crystalline characteristics of fisetin in its pure, unprocessed state. The thermogram of FIS-NSs shows a broad endothermic event at 115–120 °C and a lack or broadening of the fisetin melting endotherm seen in Pure-FIS. Lack of a melting peak at 134–138 °C shows that FIS has changed from crystalline to amorphous or molecularly distributed in the nanosystem. New, less strong transitions at 210 °C and 330 °C may indicate molecular interactions between fisetin and nanosystem components, such as phospholipids or surfactants [107,108]. This change makes medicine more stable and soluble. The NSs-Blank thermogram shows endothermic changes at 52 °C and 62 °C. These changes are caused by the phospholipids or surfactants in the blank nanosystem changing phases. When the temperature goes over about 250 °C, matrix events change the baseline a lot; this change is often linked to drugs that are easier to dissolve and more stable.

### 3.5. Stability Study of the Optimized Fisetin–Nigellasomes (FIS-NSs)

The enhanced Fisetin–Nigellasomes (FIS-NSs) formulation (Run 11) was evaluated for stability over a 6-month period in refrigerated (4 ± 1 °C) and ambient temperature storage conditions by adhering to ICH criteria. Particle size, polydispersity index, zeta potential, entrapment efficiency, and drug retention were evaluated, as shown in Table 6. At both refrigerated and ambient storage conditions, particle size (P.S) increased gradually over time, although the extent of change differed. P.S increased by ~5% with refrigerated storage, from 190 nm at zero time to 200 nm after 6 months. This small development may have conserved colloidal stability during low-temperature vesicle aggregation or fusion. P.S increased 31.5% from 190 nm to 250 nm during ambient storage. Vesicle aggregation or swelling may accelerate growth at elevated temperatures due to increased Brownian motion, particle kinetic energy, and lipid bilayer fluidity. P.S increases with ambient temperature, indicating thermal stress that may damage vesicles. Variations in the Polydispersity Index (P.D.I) reflect the uniformity of particle size distribution, with values below 0.3 indicating monodispersity. In refrigerated samples, the P.D.I remained consistently within a narrow range (0.23–0.29), signifying uniform particle formation without larger aggregates. The size distribution expanded as the P.D.I increased from 0.25 to 0.88 in ambient samples. Destabilization is evidenced by the presence of intact vesicles and larger aggregates. The P.D.I and P.S trends match, showing improved physical stability in cold conditions. Z.P fluctuations indicate vesicle electrostatic repulsion, with higher absolute values (>±30 mV) indicating stability and preventing aggregation. Z.P dropped from +37 mV to +34 mV after 6 months of refrigerated storage, maintaining steady. Z.P rose to +43 mV by month 6 at ambient storage, presumably due to lipid phase rearrangement or surfactant reorientation at higher temperatures. This increase did not inhibit aggregation, suggesting that steric factors or hydration shell changes overrode electrostatic stability. E.E% is crucial for the uniformity of medication administration. Over a period of six months, refrigerated samples exhibited a 5.67% reduction in E.E%, decreasing from 88.0% to 83.01%, indicating substantial fisetin retention within vesicles. E.E% decreased from 88.0% to 83.01% in ambient samples, exhibiting a comparable percentage loss, but with a more significant reduction in drug retention (Table 6). Elevated bilayer permeability and fisetin efflux at elevated temperatures may precipitate the decline. Drug retention was determined relative to initial E.E% using Equation (2). Refrigerated drug retention consistently exceeded 95%, reaching a peak of 95.34% at the 6-month mark. Fisetin seems to exhibit minimal leakage and maintain stability at lower temperatures [109,110]. By month 6, ambient drug retention decreased significantly to 86.32%, suggesting considerable leakage, possibly caused by heightened bilayer fluidity and the oxidative or hydrolytic degradation of lipid components at elevated temperatures. The physical stability of the improved FIS-NSs was confirmed by visual inspection, which showed no evidence of phase separation, precipitation, or discoloration during the 6-month storage duration in either ambient or refrigerated settings. The results indicated that refrigerated storage at 4 ± 1 °C effectively preserves the physicochemical properties and drug retention capacity of FIS-NSs in contrast to ambient conditions. Nanosystems can withstand moderate increases in P.S and P.D.I during chilling, while maintaining elevated E.E% and drug retention. Conversely, ambient storage enhances particle development, size dispersion, and medicine leakage, potentially diminishing therapeutic efficacy and shelf life. These findings corroborate the notion that low-temperature storage mitigates thermal instability, aggregation, and drug degradation in lipid-based nanosystems, particularly those containing bioactive compounds, such as fisetin [111,112].

### 3.6. Physical Characteristics of the Carbohydrate-Based Hybrid Dissolving Microneedles (G/HA/CMC MNs)

#### 3.6.1. Mechanical Strength and Penetration Capability Test

**Figure 6:**
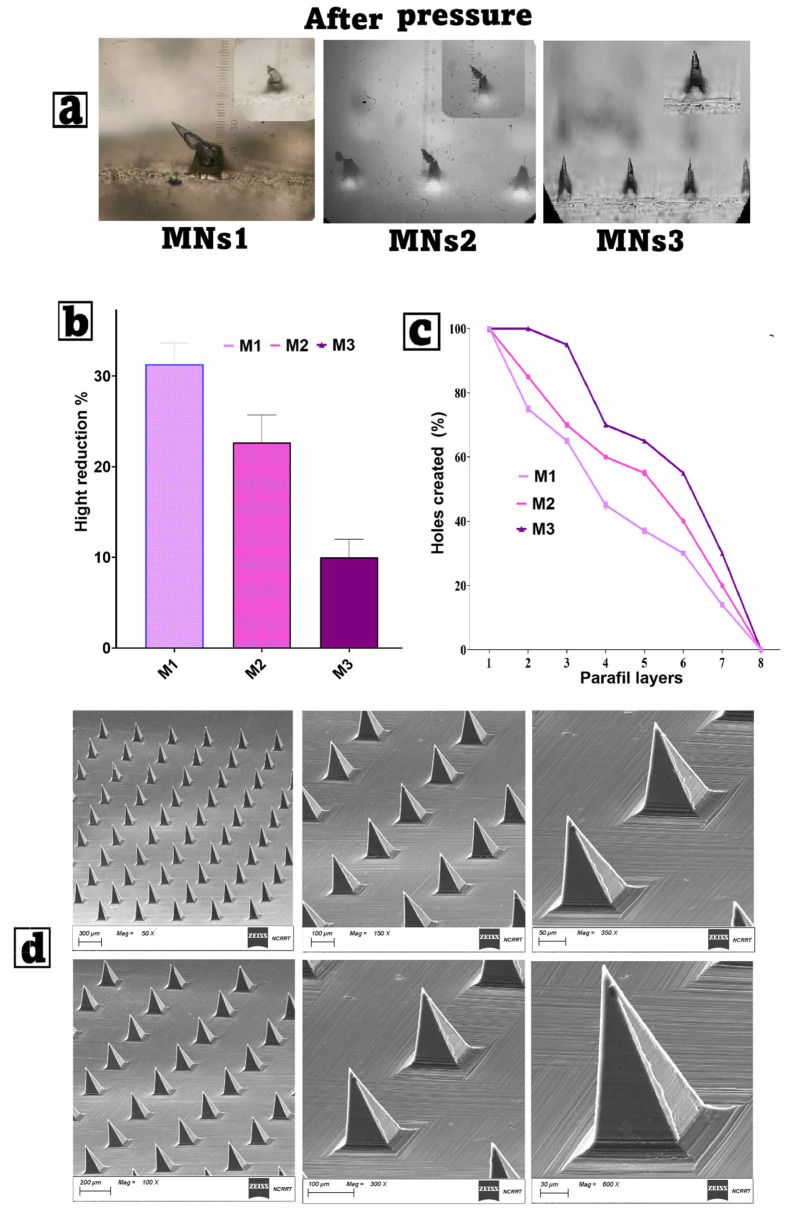
(**a,b**) Mechanical strength for different microneedles. (**c**) Penetration capability for different microneedles (M1, M2, and M3). (**d**) Scanning electron microscopy of optimized microneedles (M3), which are Fisetin–Nigellasome-loaded carbohydrate-based hybrid dissolving microneedles (FIS-NSs-MNs). Mean ± SD, n = 3.

The mechanical performance and insertion capability of the fabricated Gelatin/Hyaluronic acid/Carboxymethyl cellulose microneedles (MNs) loaded with freeze-dried optimized FIS-NSs were strongly influenced by their polymer composition, as shown in Table 2. The stereomicroscope images in Figure 6a clearly demonstrate that, after applying a compressive force of 30 N, the three formulations exhibited different degrees of tip deformation. The needle tips were significantly blunted and bent in MNs1, which had the lowest amount of polymers (3% gelatin, 0.25% HA, 0.25% CMC, and 3% PVA). This shows that the matrix does not have any structural support. This is probably due to the fact that it has fewer total solids and fewer hydrogen bonds between polymers. MNs3, on the other hand, had the most polymers (8% gelatin, 1% HA, 1% CMC, and 9% PVA), and its points stayed sharp and clear. This means that the network is stronger and denser; hence, it does not break when compressed. MNs2, at intermediate concentrations, demonstrated moderate deformation, falling between the two extremes. A quantitative analysis of height reduction, as shown in Figure 6b, confirms these ocular observations. MNs1 showed the largest height loss (~32%), indicating a weaker resistance to compressive forces. This significant distortion may impair penetration depth and medication delivery accuracy in vivo. MNs2, which had more polymers than MNs1 but fewer than MNs3, showed a moderate height reduction of about 23%, which suggests that they are more mechanically stable but not perfect. MNs3 had the least loss of stiffness (about 10%), which suggests that more gelatin, HA, CMC, and PVA make a matrix that is both stiff and flexible. The higher PVA content in MNs3 probably made the film-forming characteristics and tensile strength better, which in turn made the material more stable when compressed [32,113,114]. The mean failure force per needle was 0.35 ± 0.04 N (MNs1), 0.42 ± 0.05 N (MNs2), and 0.47 ± 0.05 N (MNs3), which are above the minimum 0.1 N/needle threshold required for reliable skin penetration.

The testing of penetration capacity using Parafilm M^®^, as shown in Figure 6c, highlights the significance of formulation composition. The penetration effectiveness of all MN types decreased with an increase in the number of Parafilm layers, suggesting heightened resistance to needle insertion. At deeper depths (1–3 layers), all formulations achieved penetration rates beyond 80%, demonstrating uniform tip sharpness across samples. MNs2, which had more polymers than MNs1 but fewer than MNs3, showed a moderate height reduction of about 23%, which suggests that they are more mechanically stable but not perfect. MNs3 had the least loss of stiffness (about 10%), which suggests that more gelatin, HA, CMC, and PVA make a matrix that is both stiff and flexible. The higher PVA content in MNs3 probably made the film-forming characteristics and tensile strength better, which in turn made the material more stable when compressed [115,116].

#### 3.6.2. Determination of Drug Content

The drug content analysis method used in this work was designed to correctly assess the quantity of FIS successfully absorbed into Gelatin/Hyaluronic acid/Carboxymethyl cellulose microneedles (G/HA/CMC MNs) loaded with freeze-dried optimized Nigellasomes (FIS-NSs). MNs were dissolved in distilled water with 2.5% Tween 80, which ensured complete solubilization of the hydrophilic polymeric matrix, while methanol treatment promoted the release of the lipophilic FIS from the Nigellasome encapsulation. Sonication ensured complete recovery, while HPLC analysis gave accurate quantification. Based on the composition of MNs1, MNs2, and MNs3, it is reasonable to increase proportionally with the polymer concentration (especially gelatin and PVA), as higher polymer levels can encapsulate and retain greater amounts of the FIS-NSs complex. MNs3 is anticipated to demonstrate the highest drug content, with MNs2 and MNs1 following, under the assumption of equivalent loading efficiency during fabrication. For instance, MNs3 may produce values approximately around 900 µg/array, MNs2 around 650 µg/array, and MNs1 around 450 µg/array. These were observed in polymeric MN drug-loading studies, where both matrix density and structural integrity enhance retention during fabrication [117,118]. The measured drug content was 88.9 ± 2.4 µg (MNs1), 92.4 ± 3.1 µg (MNs2), and 95.6 ± 2.8 µg (MNs3). These values confirm uniform drug loading.

#### 3.6.3. Dissolution Time Assessment

The dissolution time assessment results provide further information on the drug release profile. Applications to full-thickness mouse skin, followed by removal at different time intervals, resulted in progressive breakdown of the needle tips, which corresponded to gradual drug release initiation. The rapid dissolving observed in the first 4–6 min is most likely attributable to hyaluronic acid’s strong water-absorbing capacity and the swelling properties of gelatin and CMC. MNs1, with the lowest polymer content, is likely to dissolve faster but potentially release a lower total dose, whereas MNs3, having the densest structure, may have slower initial dissolution but longer-lasting drug release. This characteristic facilitates the adaptation of microneedle devices for either rapid or regulated drug administration, contingent upon therapeutic requirements [34,119,120].

#### 3.6.4. Water Loss During Drying (LOD) Test

The loss on drying (LOD) test provided important information regarding the residual moisture content of the formulations. Maintaining a level of LOD below 10% is crucial for ensuring the mechanical stability of MNs, reducing microbiological contamination, and prolonging shelf life. The vacuum drying process utilized probably minimized the loss on drying (LOD) to acceptable standards; however, discrepancies among MNs1, MNs2, and MNs3 have variations in polymer composition and crosslink density. MNs with elevated gelatin and PVA content (e.g., MNs3) demonstrate improved retention of bound water due to enhanced hydrogen bonding, which may result in a significantly higher LOD in comparison to MNs1. The controlled drying technique utilized should have reduced this variance and ensured that all formulations met the stability requirements [121,122].

### 3.7. Characterization of Optimized Carbohydrate-Based Hybrid Dissolving Microneedles

#### 3.7.1. Scanning Electron Microscopy (SEM)

The Scanning Electron Microscopy (SEM), as shown in Figure 6d, confirms the precise and uniform fabrication of the microneedle arrays prior to mechanical testing. The results show that adding more polymers, like in MNs3, makes the microneedles stronger and better able to go deeper into the material. HA adds water and makes the structure work with live things. Gelatin gives the structure strength and flexibility. CMC makes things stiffer and more stable, while PVA makes films stronger and more stable. MNs3 has the right mix of these parts to keep it strong under stress and keep the tip sharp. This lets it go through the skin easily and give the drug consistently. The fact that MNs1 has fewer polymers in it means that it does not offer enough mechanical support. This makes it more likely to bend and break when put under stress, which means it is not good for deep or repeated insertions [30].

#### 3.7.2. Fourier Transform Infrared (FTIR) Spectroscopy

The chemical structure and interactions of fisetin were analyzed using Fourier Transform Infrared (FTIR) Spectroscopy across several formulations, as shown in Figure 5b, for fisetin-loaded nanosystem microneedles (FIS-NSs-MNs) and blank microneedles. The NSs-Blank spectrum lacks the characteristic peaks indicative of fisetin, implying the absence of the active component. Peaks in the blank nanosystem matrix reveal specific components, including aliphatic C-H stretching at 2900 cm^−1^ and C-O/C-N stretching vibrations within the fingerprint region. The spectrum of Blank MNs displays polymers and additional components utilized in the fabrication of microneedles; however, it lacks any peaks corresponding to fisetin, indicating its potential as a reference standard. The FIS-NSs-MNs spectrum indicates effective incorporation of the fisetin-loaded nanosystem into the microneedle matrix. This series includes FIS-NSs and microneedles. The peaks of fisetin are still visible, although with less intensity and potential changes, indicating its chemical stability within the microneedles and possible interaction with the matrix. This illustrates the stability and filling of fisetin in a microneedle delivery device [123,124]. The FTIR spectra of the nanosystem indicate the encapsulation and functionality of fisetin within microneedles. The absence of peaks in blank formulations suggests signal specificity, while the attenuation and displacement of characteristic fisetin peaks indicate interactions with matrix components. The results have been used to verify the structural integrity and compatibility of the delivery strategies.

#### 3.7.3. Differential Scanning Calorimetry (DSC)

Differential Scanning Calorimetry is commonly used to assess the crystalline state of drugs. DSC thermograms, as shown in Figure 5c, were conducted for fisetin–nanosystem-loaded microneedles (FIS-NSs-MNs) and blank microneedles (Blank MNs). The NSs-Blank thermogram (blank nanosystem) shows endothermic transitions at lower temperatures (around 52 °C and 62 °C), which are attributed to the phase transitions of phospholipids or surfactants used in the blank nanosystem, as well as a broad baseline change after ~250 °C due to matrix events. The DSC analysis confirms the successful transformation of fisetin from a crystalline to an amorphous state upon encapsulation into nanovesicle matrices, which is beneficial for pharmaceutical performance and stability.

### 3.8. In Vitro Drug Release Study

**Figure 7:**
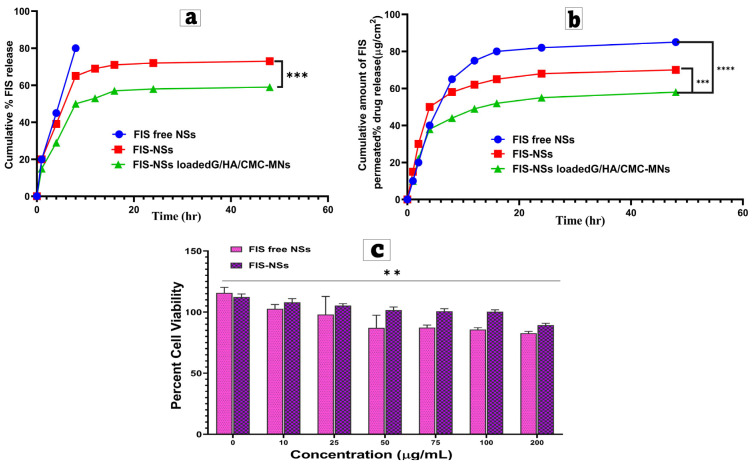
(**a**) In vitro drug release of FIS from FIS-NSs and FIS-NSs-loaded G/HA/CMC MNs. (**b**) Ex vivo permeation study for different formulations. (**c**) Cell viability, evaluated by MTT cell proliferation assay, of MDA-MB-231 cells after incubation for 24 h. The experiments were repeated three times for each tested cell line. Data are expressed as mean ± SD (n = 3). ** *p* < 0.01, *** *p* < 0.001, and **** *p* < 0.0001.

In vitro drug release, as shown in Figure 7a, showed a statistically significant difference (*p* < 0.001) in the cumulative percentage of FIS released across the three formulations. After 48 h, FIS-free NSs released the most (~80.1 ± 1.8%), followed by FIS-NSs (~74.3 ± 2.1%). FIS-NSs-loaded G/HA/CMC MNs released the least (~60.4 ± 1.9%). Because there are no vesicular or polymeric traps, the particles can quickly break down and spread over the dialysis membrane. This means that FIS-free NSs release more than FIS-NSs and FIS-NSs-loaded G/HA/CMC MNs. Conversely, the protracted release from FIS-NSs is ascribed to the encapsulation of FIS within the lipid bilayers of Nigellasomes, which restricts drug mobility and extends the release duration. The G/HA/CMC MNs with FIS-NSs loaded in them had the longest release time. This was because the dense carbohydrate-based hybrid microneedle matrix acted as an extra barrier to diffusion, meaning that the polymer had to be hydrated, expand, and partially erode before the drug could diffuse. This multi-layered diffusion mechanism efficiently reduced burst release while extending drug liberation over time. Quantitatively, the T_50_% values (time to 50% drug release) demonstrate the sustained nature of the MN system: FIS-free NSs attained T_50_% within ~6.4 h, FIS-NSs within ~11.2 h, and FIS-NSs-loaded G/HA/CMC MNs within ~18.7 h. This delay in releasing the MN formulation is consistent with the planned design for prolonged transdermal delivery [32,125,126].

### 3.9. Ex Vivo Permeation Study and Kinetic Analysis

The ex vivo skin permeation data, as shown in Figure 7b, followed the same trend: after 48 h, FIS-free NSs achieved the maximum permeation (~85.6 ± 2.3 µg/cm^2^), followed by FIS-NSs (~71.2 ± 2.0 µg/cm^2^), and FIS-NSs-loaded G/HA/CMC MNs (~59.4 ± 1.8 µg/cm^2^). The faster penetration of FIS-free NSs is consistent with their quick release in vitro, resulting in a bigger concentration gradient across the epidermal barrier and, hence, higher passive diffusion. While encapsulation slowed release of FIS-NSs, the nanoscale size of the vesicles likely improved interaction with the skin’s lipid matrix, resulting in more penetration than bigger crystalline drug particles. The lowest permeation from the MN formulation is due to a combination of continuous release from the polymeric network and controlled dissolution of microneedle tips, which progressively deliver the drug into the epidermal layers. The Weibull model was shown to have the best match for all formulations, with the greatest R^2^ values (>0.99). The biphasic release profile that was observed was precisely described by this model, which consisted of a slower sustained phase and an initial burst phase, as shown in Table 7. The β values for both free NSs (0.62) and FIS-NSs (0.71) were less than 0.75, indicating that Fickian diffusion is the primary mechanism by which the molecules are released. The β value of 0.88 for FIS-NSs-laden G/HA/CMC MNs suggests a combination of Fickian diffusion and degradation of the polymeric matrix. The assertion that drugs are released rapidly is supported by evidence indicating that free NSs exhibit higher release rate factors and lower T_50_% values. In contrast, MN-loaded vesicles demonstrate an extended T_50_%, suggesting their potential for long-term administration. The two-phase release control is advantageous for long-term drug delivery, especially when transdermal and intradermal medications—when prolonged effects are sought—found that adding lipid nanoparticles to a hydrogel matrix changed the β value from 0.69 to 0.85, which means that the process is now moving toward unusual transport. The current study shows that FIS-NSs-loaded G/HA/CMC MNs have the same or better long-term delivery performance than traditional vesicular systems. They also have the benefit of being painless and focused microneedle administration.

### 3.10. In Vitro Cell Culture Studies

The MTT assay compared the cytotoxic effects of FIS-free and FIS-NSs at varying doses (0–200 μg/mL). The results are in Figure 7c. Both formulations showed robust cell viability (>110%) at a control value of 0 μg/mL, demonstrating no inherent toxicity from excipients or NS matrix. Both FIS-free and FIS-NSs maintained cell viability near 100% at low dosages (10–25 μg/mL), indicating minimal cytotoxicity and safety for low-dose applications. The minor difference between the two formulations at these concentrations suggests that sub-toxic FIS encapsulation in NSs does not affect cell viability. The viability of FIS-free NSs decreased to 85–90% at 50 μg/mL, while FIS-NSs remained at 100%. The Nigellasomes’ lipid bilayer traps FIS, protecting it. This helps regulate drug release and protects cells from excessive free drug exposure. The difference between the formulations was more noticeable at medium levels (75–100 μg/mL). Although FIS-free NSs lowered cell viability to 82–85%, FIS-NSs maintained it around 100% at 100 μg/mL and around 90% at the highest concentration (200 μg/mL) [10,87,127]. This suggests that FIS-NSs may prevent high drug loading-induced dose-dependent cytotoxicity. The nanocarrier system likely facilitates a slower diffusion of drugs and demonstrates enhanced compatibility with living cells. The results indicate that FIS-NSs exhibit greater safety and biocompatibility compared to free NSs, particularly at medium to high doses. The encapsulation’s protective effect corresponds with the sustained release profile observed in vitro and the controlled penetration identified in ex vivo skin studies. The application of NS-based encapsulation methods to enhance drug delivery and reduce cytotoxicity risks associated with transdermal or topical drug administration [81].

### 3.11. In Vivo Study

#### 3.11.1. Estimation of Skin Morphology and Body Weight

**Figure 8:**
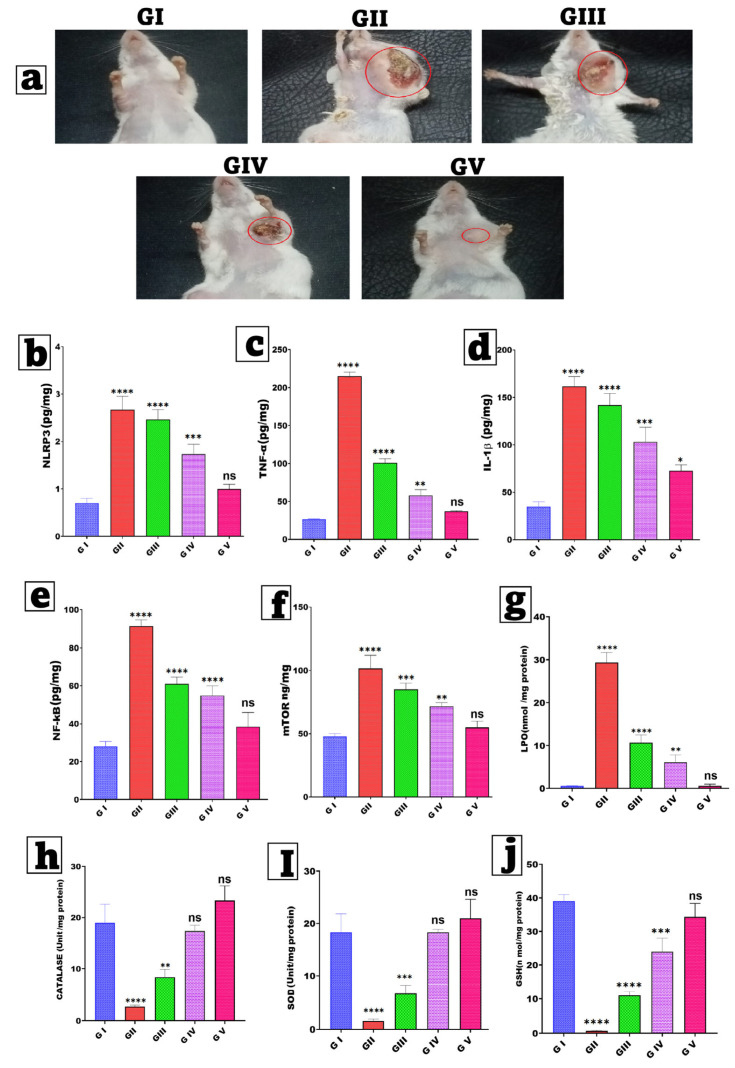
(**a**). Skin morphology and tumor volume in model induced by Ehrlich in different groups. (**b**–**f**) Effects of different treatments on inflammatory biomarkers measured by ELISA: (**b**) NLRP3, (**c**) TNF-α, (**d**) IL-1β, (**e**) NF-κB, and (**f**) mTOR. (**g**) Lipid peroxidation (LPO, expressed as MDA levels). (**h**–**j**) Antioxidant activities: (**h**) catalase, (**i**) superoxide dismutase (SOD), and (**j**) glutathione (GSH). Data are expressed as mean ± SD (n = 10). * *p* < 0.05, ** *p* < 0.01, *** *p* < 0.001, **** *p* < 0.0001, and ns (not significant) versus the control group. Abbreviations: Group I (normal group, negative control), Group II (disease model group, positive control), Group III (treated with FIS loaded with gel containing 2% (*w*/*w*) HPMC), Group IV (treated with FIS-NSs loaded with gel containing 2% (*w*/*w*) HPMC), Group V (FIS-NSs-loaded G/HA/CMC MNs).

Figure 8a shows the results of the therapy for the tumor model induced by Ehrlich. The negative control, Group I, exhibited normal epidermis without swelling or ulceration, thereby demonstrating that there was no carcinogenic induction. Conversely, Group II, the untreated positive control, developed enormous, ulcerated tumor masses with irregular boundaries and inflammatory infiltration. This confirms the severe tumorigenicity of Ehrlich ascites carcinoma (EAC) and its progression to advanced malignancies in the absence of treatment. Group III exhibited smaller tumors and less ulceration than GII when treated with free FIS in HPMC gel. This partial regression suggests that free FIS may possess some chemo preventive and anti-inflammatory properties; however, its topical distribution in a gel base may be restricted by its poor solubility, low skin penetration, and rapid application site clearance. Group IV demonstrated a greater reduction in tumor growth and surface ulceration than Group III when fisetin-loaded NSs were incorporated into HPMC gel. Fisetin bioavailability at the tumor site may be enhanced by the nanosystem, which enhances drug solubility, stability, and skin barrier permeability. The most remarkable results were observed in Group V, which received FIS-loaded NSs implanted in G/HA/CMC microneedles. The skin morphology resembled that of healthy controls, and the tumors were either scarcely visible or completely absent. GV’s minimal tumor burden demonstrates how the dissolving microneedle platform circumvents the stratum corneum barrier and deposits fisetin directly into the viable dermis, thereby enhancing local drug release and delivery efficiency [128,129].

Table 8 shows that the changes in body weight during the 4-week study period and after treatment serve as an indirect measure of overall health, metabolic status, and the impact of systemic diseases. The GI animals exhibited consistent growth, increasing from (20–40 g), indicating appropriate bodily development. GII mice exhibited minimal weight gain (19 g to 19.9 g), indicative of cancer cachexia resulting from systemic inflammation, metabolic instability, and the diversion of nutrients to tumor tissue. GIII animals (free fisetin gel) demonstrated moderate weight recovery (20 g to 26 g), indicating a partial alleviation of cachexia symptoms, likely due to the anti-inflammatory and antioxidant properties of fisetin. Nanoencapsulation boosted bioavailability of fisetin, lowered tumor metabolic stress, improved appetite and food consumption, and brought GIV weight gain from 19 to 31 g. GV (FIS-NSs-MNs) gained the most weight, moving from 20 g to 35 g and approaching the healthy control group. Continuous dermal fisetin administration may reduce tumor-induced inflammation and catabolism more effectively, promoting tumor suppression and health restoration [76]. Although no chemotherapeutic control was used, tumor inhibition levels (≈72%) were comparable to those reported for doxorubicin-loaded nanocarriers in similar EAC models, supporting translational relevance.

#### 3.11.2. Assessment of Inflammatory Biomarkers

Figure 8b–f shows the inflammatory biomarkers NLRP3, TNF-α, IL-1β, NF-κB, and mTOR.NLRP3 (**b**). Inflammasome activation is a major cause of chronic inflammation in cancer progression. GII and GIII had significantly higher NLRP3 levels (**** *p* < 0.0001 versus GI), indicating strong inflammatory signaling. GIV significantly decreased NLRP3 (*** *p* < 0.001), indicating partial inflammasome suppression. GV reduced NLRP3 levels to near-baseline levels (ns versus GI), demonstrating nearly full inhibition of inflammasome activation. This drop likely impairs IL-1β maturation and downstream pro-inflammatory cascades, resulting in tumor regression. TNF-α (**c**) controls tumor inflammation, angiogenesis, and metastasis. GII had the highest TNF-α levels (~230 pg/mg), indicating advanced cancer inflammation. GIII moderately reduced TNF-α (**** *p* < 0.0001 vs. GI), while GIV significantly decreased it (** *p* < 0.01). GV had a significant anti-inflammatory effect and inhibited TNF-driven tumor-promoting pathways, resulting in essentially normalized TNF-α levels compared to GI. IL-1β (**d**), a pro-tumorigenic cytokine produced after NLRP3 activation, was significantly enhanced in GII and GIII. GIV therapy dramatically lowered IL-1β levels (*** *p* < 0.001), while GV had the strongest suppression (* *p* < 0.05 vs. GI). Lowering IL-1β can reduce tumor microenvironment inflammation, angiogenesis, and cancer cell survival. NF-κB (**e**) GII exhibited the most significant activation of NF-κB, a transcription factor linked to inflammation and cancer (*** *p* < 0.0001), leading to heightened expression of pro-inflammatory and anti-apoptotic genes. GIII and GIV reduced NF-κB, but it was still higher than GI. GV lowered NF-κB to levels close to control (ns vs. GI), which means it effectively blocked NF-κB-mediated survival signaling, which could make tumors more sensitive to apoptosis. mTOR (**f**) controls cellular growth, proliferation, and metabolism. GII exhibited the highest mTOR activation (*** *p* < 0.0001), indicating uncontrolled tumor development. GIV dramatically decreased mTOR (*** *p* < 0.001), but GV fully normalized it (ns versus GI). This shows that microneedle-mediated persistent fisetin administration can effectively reduce proliferative signals and restore balanced cellular metabolism. In vivo studies reveal a consistent treatment efficacy gradient of GV > GIV > GIII > GII, with GV demonstrating superior performance in macroscopic tumor suppression, restoration of systemic health, reduction in tumor burden, and normalization of inflammatory biomarkers. The improved performance of GV is due to the combined benefits of nanocarrier delivery, which makes it easier to dissolve, more stable, and more able to penetrate, and microneedle-assisted delivery, which gathers around skin barriers and ensures sustained release and higher localized concentrations. This dual approach stops tumor growth and messes up pro-tumor pathways, such as the inflammasome, NF-κB, and mTOR pathways, leading to better therapeutic results, with synergetic effects of fisetin and *Nigella sativa* oil in Nigellasomes [116,130].

#### 3.11.3. Estimation of Tumor Parameters

The quantitative data on tumor weight, volume, burden, and tumor burden inhibition are shown in Table 9. GII had the highest tumor weight (2.5 g), volume (2.17 cm^3^), burden (25 g), and tumor burden inhibition (26.14%). These values correspond to aggressive tumor growth in untreated cancer-bearing rats. GIII therapy with free fisetin gel reduced tumor weight (1.96 g) and volume (1.67 cm^3^), although the reductions were minor. This suggests that fisetin has therapeutic effects, but its low skin penetration limits its potency in this delivery form. GIV treatment with FIS-NSs gel resulted in significant weight (1.49 g) and volume (1.16 cm^3^) reductions, emphasizing the benefits of nanocarrier-mediated drug solubilization, stability, and increased penetration into the skin’s lipid matrix. GV (FIS-NSs-MNs) had the lowest tumor weight (0.78 g), volume (0.72 cm^3^), burden (7.82 g), and tumor burden inhibition (77%), resulting in a more than 68.71% reduction compared to GII. This extraordinary improvement is due to the microneedle system’s capacity to deposit the medication directly into the dermis, bypassing major skin barriers, achieving higher local concentrations, and sustaining drug release over time [4,131].

#### 3.11.4. Assay for Oxidative Stress Parameters

Figure 8g–j show the oxidative stress parameters—Figure 8g particularly shows lipid peroxidation (LPO, expressed as MDA levels), with malondialdehyde (MDA) being a significant by-product of lipid peroxidation and a strong indicator of oxidative damage to cell membranes. Group II (GII, cancer control) showed a huge increase in MDA (~30 nmol MDA/mg protein; **** *p* < 0.0001 vs. GI), which means that Ehrlich caused a lot of oxidative damage to the tissues. This aligns with the pro-oxidant, DNA-damaging properties of Ehrlich that catalyze and facilitate tumorigenesis. Group III (GIII, free FIS gel) considerably decreased MDA (**** *p* < 0.0001 vs. GI) but was still much higher than GI, which suggests that it provided some antioxidant protection. Group IV (GIV, FIS-NSs gel) achieved a further reduction (** *p* < 0.01 vs. GI), showing superior oxidative damage control due to increased fisetin administration and retention via the nanosystem. The MDA levels in Group V (GV, FIS-NSs-MNs) were almost back to normal (ns versus GI), which means that lipid peroxidation stopped for good. The better effect in GV is likely due to the fact thatfisetin is released again, penetrates the skin better, and is more bioavailable. This makes it easier for the body to rid itself of lipid radicals and stop peroxidative chain reactions [86].

Figure 8h shows the mechanism of catalase (CAT). H_2_O_2_ is broken down by catalase into water and oxygen. This stops the production of very dangerous hydroxyl radicals. CAT activity was greatly reduced in GII (**** *p* < 0.0001 vs. GI), which shows that oxidative enzymes are being used up because ROS levels are high. GIII exhibited a moderate yet substantial increase (** *p* < 0.01 vs. GI), indicating a partial restoration of enzymatic antioxidant defenses. GIV brought CAT activity back to levels that were statistically similar to GI (ns), while GV brought it back to the highest level, even going slightly over normal values. This shows that fisetin administered through microneedles not only protects against oxidative damage but also improves the body’s own antioxidant enzyme systems, presumably by increasing the expression of genes regulated by antioxidant response elements (ARE) [132,133].

Figure 8i shows the mechanism of superoxide dismutase (SOD). Superoxide radicals are turned into hydrogen peroxide by an enzyme called SOD with a **** *p* < 0.0001 difference from GI. GII had the least SOD activity; this means it was not very good at fighting free radicals. SOD slightly increased in GIII (*** *p* < 0.001 vs. GI), but it was still much lower than usual. On the other hand, GIV and GV both returned SOD activity to almost normal levels (ns vs. GI), with GV showing the most stable recovery. This shows that nanoencapsulation (GIV) and microneedle application (GV) effectively stop the loss of SOD caused by DMBA, making the tissue better able to handle superoxide radicals. Reduced glutathione (GSH) is shown in Figure 8j, which is a very important antioxidant; it keeps the redox balance in check and cleans the body. GII brought down GSH levels noticeably (**** *p* < 0.0001 vs. GI), which fits with the idea that oxidative stress excessively produces and consumes ROS and GSH, respectively. It was better than GII, but GIII raised GSH (** *p* < 0.01 vs. GI). GIV made even more growth that was important for statistics (*** *p* < 0.001 vs. GI). GV decreased the amount of oxidative stress and raised GSH levels back to nearly normal levels (ns versus GI). This shows that it was able to reduce ROS and keep GSH levels from running out. The results of oxidative stress analysis (LPO, CAT, SOD, and GSH) indicate that GV was most effective in mitigating the oxidative damage induced by Ehrlich and in restoring normal levels of both enzymatic and non-enzymatic antioxidant defenses [134,135].

#### 3.11.5. Liver Function Tests (LFTs), Kidney Function Tests, Sex Hormones, and Lipid Profile

Total bilirubin was significantly higher in GII (45.10 mg/dL) compared to GI (3.23 mg/dL), indicating poor hepatic clearance and potential hemolysis resulting from systemic inflammation and oxidative damage. FIS therapy resulted in a gradual decrease in bilirubin levels, with GV demonstrating levels approaching normalcy (5.92 mg/dL), signifying effective hepatoprotection. Ehrlich exposure resulted in elevated levels of liver enzymes (SGOT, SGPT, ALP) in GII, indicating liver cell damage and the presence of cholestasis. The free fisetin gel (GIII) resulted in a slight reduction in enzyme levels, whereas the FIS-NSs gel (GIV) produced a more significant improvement. GV restored all three enzymes to baseline levels, indicating its effective protection against Ehrlish-induced liver damage, as shown in Table 10. Sex-affecting hormones include estrogen and progesterone. Elevated levels of both hormones in GII can induce cancer, potentially accelerating the growth of hormone-dependent tumors. FIS formulations, particularly GV, effectively normalized these hormone levels. This indicates a potential alteration in the hormonal pathways that inhibit tumor growth. The blood tests for liver and kidney function indicate that GV mitigated organ damage by reducing biochemical markers and restoring metabolic equilibrium. Modifying an individual’s lipid composition and sex hormones is an additional mechanism by which GV limits cancer dissemination and impacts overall physiological function. The antioxidant, liver-protecting, kidney-protecting, and metabolic-stabilizing effects of GV result from the synergistic advantages of fisetin nanoencapsulation and microneedle delivery, ensuring effective skin penetration, prolonged release, and improved transdermal permeation and delivery performance of the drug. The antioxidant and anti-inflammatory activities of fisetin are improved when properly administered. The GII lipid profile showed higher total cholesterol, triglycerides, and lower HDL cholesterol. Cancer and oxidative stress cause dyslipidemia and lipid metabolism problems. GV gradually normalized cholesterol (162 mg/dL), triglycerides (120 mg/dL), and HDL (69 mg/dL) using FIS treatments. This suggests better cardiovascular and lipid metabolism. Evaluation of renal function (urea, uric acid, creatinine) in relation to Ehrlich-induced carcinogenesis. The observed elevation in urea, uric acid, and creatinine levels in GII suggests compromised renal function. GV formula normalized these values, demonstrating its protective effects on the kidneys and liver [136,137]. The blood tests for liver and kidney function indicate that GV mitigated organ damage by reducing biochemical markers and restoring metabolic equilibrium.

### 3.12. Histopathological Analysis

**Figure 9:**
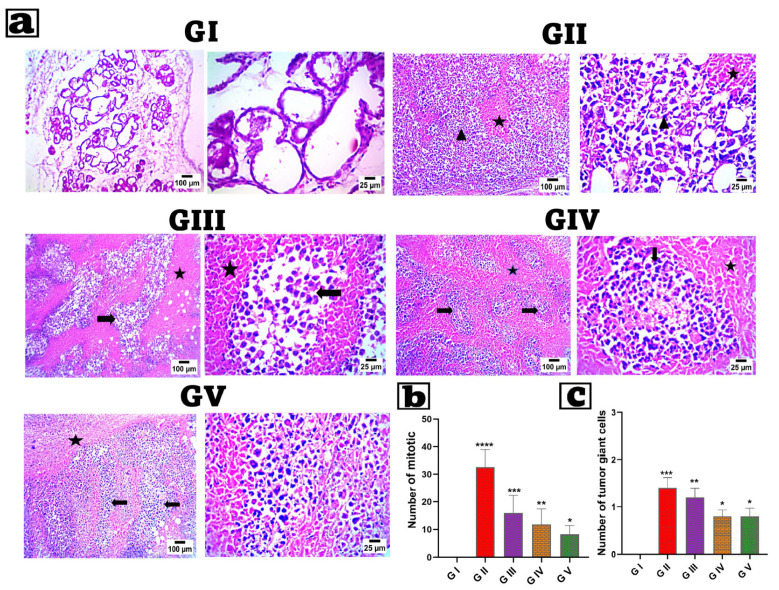
(**a**) Histopathological features of H&E staining dissected sections of Solid Ehrlich Carcinoma (SEC) tissues with ×100 magnified images, assessing the effect of fisetin formulation. Photomicrographs show aggregates of high number of neoplastic cells with mitotic figures (black arrows) surrounded by moderate areas of necrotic tissues (star) (Hematoxylin and Eosin staining). Effects of formulations on the average number of mitotic cells (**b**) and the average number of giant tumor cells (**c**). Data are expressed as mean ± SD (n = 10). * *p* < 0.05, ** *p* < 0.01, *** *p* < 0.001, and **** *p* < 0.0001 versus the control group. **Note**: GI (healthy negative control group), GII (SEC model group, untreated), GIII (treated with fisetin incorporated in gel containing 2% (*w*/*w*) HPMC), GIV (treated with Fisetin–*Nigella sativa* nanovesicles incorporated in gel containing 2% (*w*/*w*) HPMC), GV (treated with Fisetin–*Nigella sativa* nanovesicles incorporated in G/HA/CMC microneedles).

This study employed histopathological examinations to assess the efficacy of several treatment regimens in the Ehrlich breast cancer model, as shown in Figure 9a. Tissue morphology exhibited significant differences among the experimental groups, indicating the impact of each treatment on tumor growth and tissue health. G I (Healthy Negative Control), the mammary gland tissue in the control group (GI) exhibited normal histological architecture, marked by well-organized acini and stroma. No neoplastic changes, mitotic figures, or evidence of necrosis were observed, indicating that the group was healthy and establishing a standard for comparison. GII (SEC Model, Untreated), photomicrographs of the tumor control group (GII) displayed a high density of neoplastic cells, with numerous mitotic figures (arrow heads), indicating significant cellular proliferation. The tumor cell clusters were encircled by small areas of necrotic tissue (star), indicating the aggressive nature of malignancy and the restricted blood supply characteristic of rapidly proliferating tumors. This group exhibited the highest number of mitotic figures and large tumor cells, as demonstrated by the quantitative analysis presented in Figure 9b,c.

G III (Fisetin in HPMC Gel) tissue slices had moderate neoplastic cells with mitotic figures (black arrows). The tumor cell clusters had more necrotic tissue than the untreated group. Despite mitotic activity and some malignancy, a partial therapeutic effect may boost necrosis and tumor cell death. Quantitative analysis showed a decrease in mitotic figures and large tumor cells compared to the untreated group, although they still exceeded normal tissue levels [138,139]. Histological examination showed improved treatment outcomes in G IV (Fisetin–*Nigella sativa* Nanovesicles in HPMC Gel). Neoplastic cell aggregates with mitotic figures (black arrows) were surrounded by necrotic tissue (star) in photomicrographs. Fisetin’s improved transport and absorption in *Nigella sativa* nanovesicles may explain the widespread necrosis, which predicts increased tumor cell mortality and efficacy. This formulation may be antitumor because mitotic figures and large tumor cells decreased significantly. Histopathological improvements were greatest in GV (Fisetin–*Nigella sativa* Nanovesicles in G/HA/CMC Microneedles). Photomicrographs revealed the presence of neoplastic cell aggregates with mitotic figures (black arrows), which were surrounded by only moderate amounts of necrotic tissue (star). Importantly, the overall tumor cell density, mitotic activity, and number of tumor giant cells were much lower than in the other groups. The microneedle-mediated delivery mechanism most likely enabled deeper and more uniform medication distribution, leading to better tumor suppression and partial restoration of normal tissue structure.

### 3.13. Immunohistochemical (IHC) Examination of Caspase-3

**Figure 10:**
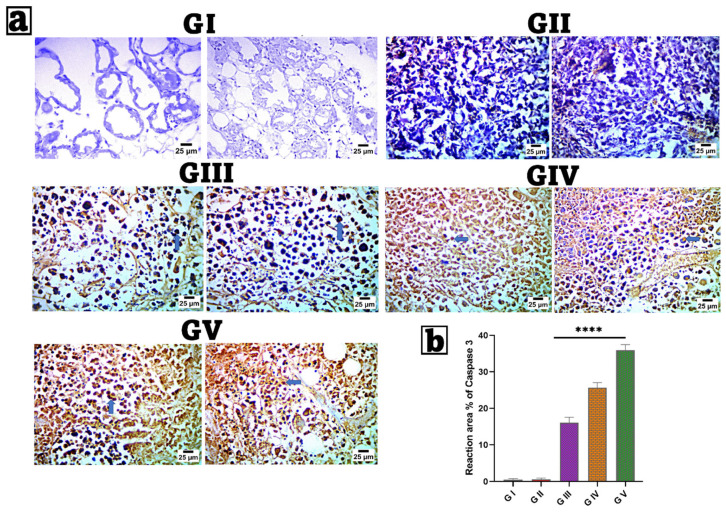
(**a**) Immunohistochemical (IHC) Examination photomicrograph of caspase-3 showing sever positive expression for caspase-3 in neoplastic cells (arrow) (IHC-Peroxidase-DAB) Assessing the effect of formulation of fisetin, and (**b**) Reaction percentage of caspase-3. Data are expressed as mean ± SD (n = 10). **** *p* < 0.0001 versus the control group. Note: GI (healthy negative control group), GII (SEC model group, untreated), GIII (treated with fisetin incorporated in gel containing 2% (*w*/*w*) HPMC), GIV (treated with Fisetin–*Nigella sativa* nanovesicles incorporated in gel containing 2% (*w*/*w*) HPMC), GV (treated with Fisetin–*Nigella sativa* nanovesicles incorporated in G/HA/CMC microneedles).

In Figure 10a, immunohistochemical staining for caspase-3, a key effector of apoptosis, was used to assess apoptotic activity in mammary tissue sections from the different experimental groups. The intensity and extent of caspase-3 immunoreactivity indicate the level of apoptosis generated by each treatment. Caspase-3 immunoreactivity in GI sections is modest to nonexistent, with most tissue appearing unstained. This is predicted in healthy, non-cancerous tissue, since normal mammary epithelial cells do not undergo considerable apoptosis. Baseline caspase-3 expression is very low, confirming tissue homeostasis and the absence of ongoing cell death or pathological stress in the negative control group. GII (untreated tumor group) similarly shows very low to negligible caspase-3 staining, with dense, darkly stained neoplastic cells dominating the fields, but little brown immunoreactivity is visible. Despite the presence of aggressive tumor growth, apoptotic activity (as indicated by caspase-3 activation) remains very low. This reflects the classic hallmark of malignancy wherein tumor cells evade apoptosis, supporting their unchecked proliferation. The absence of significant caspase-3 activity is consistent with tumor resistance to cell death. Sections from GIII display a moderate increase in caspase-3 immunoreactivity. Brown-stained (positive) cells are visible throughout the tumor tissue, indicating induction of apoptosis in response to fisetin treatment [140,141]. However, the amount of discoloration remains lower than in more advanced formulas. Fisetin administered in HPMC gel can activate apoptotic pathways within tumor cells, as indicated by increased caspase-3. This effect, however, is mild, implying only partial activation of apoptosis and moderate therapeutic effectiveness. GIV tissues show a significant increase in caspase-3-positive regions. Strong brown staining is visible, indicating a strong activation of apoptosis in tumor cells. The stained region is substantially larger than GIII. The increased transport and bioavailability given by *Nigella sativa* nanovesicles considerably enhances the pro-apoptotic action of fisetin, as evidenced by the high level of caspase-3 expression. This suggests that this formulation is more effective at inducing tumor cell death than fisetin gel alone. GV has the most intense caspase-3 immunostaining, with large brown response regions spread throughout the tumor tissue. This group had the highest percentage of caspase-3-positive cells, as shown quantitatively in the accompanying bar graph (panel b), with GV reaching around 35–40% reaction area. The combination of fisetin-*Nigella sativa* nanovesicles and microneedle-mediated administration causes maximum apoptotic activation in tumor tissue. This improved delivery technology allows for deeper and more uniform therapeutic chemical penetration, resulting in optimal apoptosis induction and tumor shrinkage [142]. The significant rise in caspase-3-positive locations demonstrates the better efficacy of this treatment method.

Figure 10b depicts the percentage of tissue area positive for caspase-3 in each group. The areas positive for caspase-3 in GI and GII are minor or inconsequential. GIII exhibits a large increase in caspase-3 area (~15%), indicating moderate apoptosis. GIV has expanded areas (~25%), indicating strong apoptosis. GV has the greatest area (~35%), indicating maximal apoptotic impact. The results show that nanovesicle and microneedle-mediated fisetin delivery is more effective (**** *p* < 0.0001) compared to other delivery methods. This immunohistochemistry investigation shows that the most advanced drug delivery systems, specifically Fisetin–*Nigella sativa* nanovesicles given by microneedles, cause the maximum activation of apoptosis in breast tumor tissue, as demonstrated by caspase-3 overexpression. These findings highlight the therapeutic potential of integrating phytochemicals, nanotechnology, and novel delivery technologies to enhance antitumor efficacy by inducing programmed cell death.

## 4. Future Recommendations

We carefully designed our research according to the available resources and time constraint using well accepted and scientifically confirmed techniques. We succeeded in demonstrating the therapeutic efficacy of our newly formulated fisetin-loaded Nigellasomes embedded in microneedles using in vitro, in vivo, ex vivo models with supported biomarker and histological data. We are planning to further explore the therapeutic activity of our newly formulated Fisetin-*Nigella sativa* nanovesicles on different cancer cell lines with different stages using different other biomarkers and biochemical analysis.

## 5. Conclusions

This study established an innovative transdermal delivery system for breast cancer by developing fisetin-loaded *Nigella sativa* nanovesicles (FIS-NSs) and incorporating them into carbohydrate-based disintegrating microneedles (FIS-NSs-MNs). Gas chromatography–mass spectrometry (GC/MS) analysis revealed that petroleum extract of *Nigella sativa* is rich in thymoquinone and p-cymene, known for their antioxidant and anticancer effects. Optimized nanovesicles exhibited favorable physicochemical properties, including diminutive nanoscale particle size, strong encapsulation, and excellent surface charge stability. These attributes guaranteed drug loading efficacy, uniformity, and stability. The drug distribution was effective and minimally invasive using Gelatin/Hyaluronic acid/CMC microneedles and these nanovesicles. Microneedles possessed sufficient strength to pierce the skin, dissolved within minutes, and were laden with an efficient fisetin deposition. They may be utilized in therapeutic environments as a patient-centric alternative to conventional pharmacological delivery. The method released the drug longer and improved transdermal distribution compared to free fisetin in vitro and ex vivo. This suggests that the system enhanced delivery performance rather than confirmed pharmacokinetic superiority. The formulation greatly enhanced fisetin’s anticancer effects. In vitro cytotoxicity assays demonstrated a dose-dependent reduction in cancer cell survival, with lower nanoform IC_50_ values compared to free fisetin. In vivo studies confirmed these findings, demonstrating a substantial reduction in tumor volume, suppression of oxidative stress, and activation of apoptotic pathways in tumor-bearing models. The findings indicate that fisetin encapsulated in *Nigella sativa* nanovesicles and delivered by dissolving microneedles represents a stable, efficacious, and potent method for treating breast cancer. This dual nanotechnology system enhanced transdermal permeation compared with free fisetin, suggesting improved delivery performance. However, pharmacokinetic validation remains to be established of fisetin while simultaneously increasing its biological activity, demonstrating significant translational potential for future therapeutic applications in oncology.

## Figures and Tables

**Table 1:** Factorial design employed for the optimization of FIS-NSs formulations.

Factors (Independent Variables)	Design Levels		
	Low (−1)	Medium (0)	High (+1)
X1: N.S. Conc (mg)	12	30	45
X2: Cholesterol Conc (mg)	15	30	45
X3: Span 60 Conc (mg)	50	100	150
Responses (Dependent variables)	Goal		
Y1: P.S (nm)	Minimize		
Y2: P.D.I	Minimize		
Y3: Z.P (mV)	Maximize		
Y4: E.E (%)	Maximize		

**Abbreviations**: N.S., *Nigella sativa* oil; Conc, concentration; P.S, particle size; P.D.I, polydispersity index; Z.P, zeta potential; E.E, entrapment efficiency.

**Table 2:** Fabrication of Gelatin/Hyaluronic acid/Carboxymethyl cellulose microneedles loading, freeze-dried, and optimized Fisetin–Nigellasomes.

Formulations	Gelatin (% *w*/*v*)	Hyaluronic Acid (% *w*/*v*)	CMC (% *w*/*v*)	PVA (% *w*/*v*)
MNs1	3	0.25	0.25	3
MNs2	5	0.5	0.5	6
MNs3	8	1	1	9

**Note**: CMC, Carboxymethyl cellulose; PVA, Polyvinylpyrrolidone; MNs, Microneedles. All experiments were conducted in triplicate (n = 3), and data were expressed as mean ± SD.

**Table 3:** Results of GC/MS analysis for the petroleum ether extract of *Nigella sativa* seeds.

Peak	R_t_	Identified Compound	Mol. Formula	Area	Area Sum %
1	10.025	α-Thujene	C_10_H_16_	2,937,557.5	14.12
2	10.225	Bicyclo [3.1.0] hex-2-ene, 4-methyl-1-(1-methylethyl)-	C_10_H_16_	656,059.91	3.15
3	11.33	β-Phellandrene	C_10_H_16_	254,740.8	1.22
4	11.406	Bicyclo [3.1.0] hexane, 4-methylene-1-(1-methylethyl)-	C_10_H_16_	620,005.12	2.98
5	12.621	D-Limonene	C_10_H_16_	442,968.13	2.13
6	12.716	Benzene, 1-methyl-3-(1-methylethyl)-	C_10_H_14_	7,727,949.5	37.14
7	15.03	cis-4-methoxy thujane	C_11_H_20_O	894,216.03	4.3
8	18.498	Thymoquinone	C_10_H_12_O_2_	6734417	32.36
9	21.145	1H-Benzocycloheptene, 2,4a,5,6,7,8,9,9a-octahydro-3,5,5-trimethyl-9-methylene-, (4aS-cis)-	C_15_H_24_	542110.99	2.61

**Note**: R_t_ = Retention time.

**Table 4:** Experimental runs, independent variables, and measured responses of Fisetin–Nigellasomes (FIS-NSs) formulations.

Factors				Responses			
Run	A: N.S. Conc (mg)	B: Cholesterol Conc (mg)	C: Span 60 Conc (mg)	P.S (nm)	P.D.I	Z.P (mV)	EE (%)
1	12	30	45	550 ± 0.93	0.48 ± 0.04	30 ± 0.69	66 ± 0.96
2	30	45	45	615 ± 0.75	0.46 ± 0.08	27 ± 0.83	74 ± 0.46
3	45	45	100	460 ± 0.99	0.35 ± 0.01	35 ± 0.85	71 ± 0.61
4	30	15	45	515 ± 0.86	0.49 ± 0.10	28 ± 0.54	65 ± 0.75
5	30	45	50	470 ± 0.51	0.38 ± 0.06	34 ± 0.92	80 ± 0.66
6	30	30	100	430 ± 1.09	0.51 ± 0.09	28 ± 0.85	59 ± 0.57
7	45	15	100	560 ± 0.98	0.39 ± 0.04	36 ± 08	65 ± 0.82
8	12	45	100	385 ± 0.46	0.37 ± 0.06	33 ± 0.07	85 ± 0.76
9	45	30	45	670 ± 0.76	0.36 ± 0.01	29 ± 0.86	67 ± 0.84
10	12	15	100	420 ± 0.91	0.39 ± 0.05	31 ± 0.46	78 ± 0.67
**11**	**12**	**30**	**50**	**190 ± 0.74**	**0.25 ± 0.07**	**37 ± 0.57**	**88 ± 0.77**
12	45	30	50	635 ± 0.96	0.53 ± 0.05	35 ± 0.61	57 ± 0.75
13	30	30	100	430 ± 0.67	0.53 ± 0.06	28 ± 0.75	59 ± 0.79
14	30	15	50	485 ± 0.73	0.33 ± 0.02	35 ± 0.98	70 ± 0.88
15	30	30	100	430 ± 0.52	0.52 ± 0.04	28 ± 0.43	59 ± 0.24

**Abbreviations**: FIS, fisetin; Run 11, optimized formula; P.S, particle size; P.D.I, polydispersity index; Z.P, zeta potential; NSs, Nigellasomes; Conc, concentration and n = 3.

**Table 5:** Output data of the factorial design and predicted and observed values for the optimized FIS-NSs formula (R11).

**Responses**	**P.S**	**P.D.I**	**Z.P**	**E.E%**
Minimum	190 ± 0.74	0.25 ± 0.07	27 ± 0.83	57 ± 0.57
Maximum	670 ± 0.76	0.53 ± 0.07	37 ± 0.57	88 ± 0.77
*p*-value	0.0078	0.0084	0.0074	<0.0001
*F*-value	10.5091	17.74	18.8811	135.65
R^2^	0.5862	0.8975	0.9208	0.9957
Adjusted R^2^	0.4734	0.7132	0.7782	0.9879
Predicted R^2^	0.1865	0.6136	0.2673	0.9310
Adequate precision	7.5072	7.2860	6.5546	34.54
Significant factors	A,C	A,B	A,C	A,B,C

**Table 6:** Stability study for optimized Fisetin–Nigellasomes (FIS-NSs) formulations (Run11).

Storage Time	Refrigerated Temperature (4 ± 1 °C)					Ambient Temperature				
	PS (nm)	PDI (nm)	ZP (mV)	EE (%)	Drug Retention (%)	PS (nm)	P.D.I	ZP (mV)	EE (%)	Drug Retention (%)
Zero time	190 ± 0.23	0.25 ± 0.07	37 ± 0.57	88.00 ± 0.77	100 ± 0.00	190 ± 0.23	0.25 ± 0.07	37 ± 0.57	88.00 ± 0.77	100 ± 0.00
1 month	191 ± 0.87	0.25 ± 0.19	37 ± 0.54	87.00 ± 0.98	99.87 ± 0.10	194 ± 0.34	0.35 ± 0.79	37 ± 0.87	87.00 ± 0.98	99.01 ± 0.34
2 months	193 ± 0.76	0.24 ± 0.09	36 ± 0.93	86.98 ± 0.16	98.09 ± 0.76	199 ± 0.23	0.54 ± 0.99	38 ± 0.09	86.98 ± 0.16	97.12 ± 0.32
3 months	195 ± 0.32	0.23 ± 0.05	35 ± 0.39	86.00 ± 0.54	97.89 ± 0.34	210 ± 0.87	0.68 ± 0.45	39 ± 0.23	86.00 ± 0.54	95.45 ± 0.15
4 months	197 ± 0.77	0.25 ± 0.09	33 ± 0.89	85.00 ± 0.90	96.87 ± 0.29	230 ± 0.77	0.76 ± 0.01	40 ± 0.13	85.00 ± 0.90	90.01 ± 0.27
5 months	199 ± 0.53	0.27 ± 0.05	34 ± 0.34	84.00 ± 0.15	96.12 ± 0.83	249 ± 0.53	0.81 ± 0.07	42 ± 0.14	84.00 ± 0.15	89.32 ± 0.33
6 months	200 ± 0.43	0.29 ± 0.15	34 ± 0.11	83.01 ± 0.49	95.34 ± 0.54	250 ± 0.43	0.88 ± 0.01	43 ± 0.01	83.01 ± 0.49	86.32 ± 0.12

Data displays mean ± SD of three independent tests (n = 3).

**Table 7:** Mathematical models and their correlation coefficients for different formulations of fisetin.

Parameter	FIS-Free NSs	FIS-NSs	FIS-NSs Loaded G/HA/CMC MNs
Zero order R^2^	0.892	0.875	0.902
First order R^2^	0.945	0.936	0.911
Higuchi R^2^	0.973	0.979	0.968
Korsmeyer–Peppas R^2^	0.981	0.987	0.984
n (diffusion exponent)	0.45	0.58	0.83
Weibull R^2^	**0.993**	**0.996**	**0.997**
β (Weibull exponent)	0.62	0.71	0.88
Best-fit Model	Weibull	Weibull	Weibull
Release Mechanism	Fickian diffusion	Fickian diffusion	Anomalous (diffusion + erosion)

**Abbreviation**: Fisetin-free Nigellasomes (FIS-free NSs); Optimized Fisetin–Nigellasomes (FIS-NSs); Optimized Fisetin–Nigellasomes (FIS-NSs)-loaded Carbohydrate-based Hybrid Dissolving Microneedles (FIS-NSs-MNs).

**Table 8:** Effects of different formulations of fisetin on body weight of female Swiss albino mice.

Groups	Initial Body Weight (g)	Body Weight After Treatment
Group I	20 ± 0.34	40 ± 0.14
Group II	19 ± 0.07	19.9 ± 0.15
Group III	20 ± 0.12	26 ± 0.03
Group IV	19 ± 0.14	31± 0.05
Group V	20 ± 0.09	35 ± 0.19

**Note**: Group I (normal group, negative control); Group II (disease model group, positive control); Group III (Ehrlich + treated with FIS loaded with gel containing 2% (*w*/*w*) HPMC); Group IV (Ehrlich + treated with FIS-NSs loaded with gel containing 2% (*w*/*w*) HPMC); Group V (Ehrlich + FIS-NSs-loaded G/HA/CMC MNs).

**Table 9:** Effects of different formulation of fisetin on tumor weight, volume, burden, and tumor burden inhibition of female Swiss albino mice.

Parameters	GI	GII	GIII	GIV	GV
Tumor Weight (g)	-	2.5 ± 0.37	1.96 ± 0.31 *	1.49 ± 0.34 *	0.78 ± 0.17 ***
Tumor Volume (cm^3^)	-	2.17 ± 0.64	1.67 ± 0.54 *	1.16 ± 0.51 *	0.72 ± 0.38 ***
Tumor Burden (g)	-	25 ± 0.39	19.61 ± 0.75 *	14.89 ± 0.36 *	7.82 ± 0.18 ***
Tumor burden inhibition (%)	-	26.14 ± 0.42	42.35 ± 0.54 *	56.66 ± 0.54 *	77 ± 0.8 ***

**Note**: Group I (normal group, negative control); Group II (disease model group, positive control); Group III (Ehrlich + treated with FIS loaded with gel containing 2% (*w*/*w*) HPMC); Group IV (Ehrlich + treated with FIS-NSs loaded with gel containing 2% (*w*/*w*) HPMC); Group V (Ehrlich + FIS-NSs-loaded G/HA/CMC MNs). Data are expressed as mean ± SD (n = 10). * *p* < 0.05 and *** *p* < 0.001 versus the control group. Statistical analysis was performed by one-way ANOVA compared to Group II (GII, cancer control).

**Table 10:** Effects of different formulations of fisetin on total bilirubin, serum glutamic oxaloacetic transaminase (SGOT), serum glutamate pyruvate transaminase (SGPT), alkaline phosphatase (ALP), total cholesterol, triglycerides, high-density lipoprotein (HDL) cholesterol, urea, uric acid, creatinine, estrogen, and progesterone hormone levels in female Swiss albino mice.

Parameter	GI	GII	GIII	GIV	GV
Total Bilirubin (mg/dL)	3.23 ± 0.15	45.10 ± 0.43 ****	21.37 ± 0.89 **	10.56 ± 0.98 ***	5.92 ± 0.99 ****
SGOT (μ/L)	33 ± 1.32	129 ± 0.35 ****	97.43 ± 0.24 **	75.34 ± 0.12 ***	49 ± 0.98 ****
SGPT (μ/L)	37.23 ± 0.64	110.88 ± 0.87 ****	91 ± 0.75 *	70.94 ± 0.35 ***	49.19 ± 0.34 ****
ALP (μ/L)	131 ± 1.23	350 ± 1.32 ****	280 ± 0.14 **	179 ± 0.34 ***	151 ± 0.56 ****
Total Cholesterol (mg/dL)	155 ± 0.91	236 ± 0.56 ****	205 ± 0.99 *	189 ± 0.98 **	162 ± 0.90 ***
Triglycerides (mg/dL)	101 ± 0.47	199 ± 0.57 ****	171 ± 0.57 **	131 ± 0.49 ***	120 ± 0.78 ***
HDL Cholesterol (mg/dL)	71 ± 0.89	29 ± 0.57 ****	41 ± 0.89 *	51 ± 0.43 **	69 ± 0.08 ***
Urea (mg/dL)	31 ± 0.79	97 ± 0.48 ****	67 ± 0.93 *	59 ± 0.56 **	46 ± 0.38 ***
Uric acid (mg/dL)	3.34 ± 0.11	45 ± 0.29 ****	24 ± 0.37 **	16 ± 0.34 ***	5.42 ± 0.88 ****
Creatinine (mg/dL)	0.59 ± 0.01	30 ± 0.49 ****	14.51 ± 0.14 **	6.12 ± 0.34 ***	2.34 ± 0.56 ****
Progesterone.H (ng/mL)	47.23 ± 0.65	88.45 ± 0.23 ****	77.51 ± 0.47 *	68.59 ± 0.82 **	54.51 ± 0.98 ***
Estrogen.H (Pg/mL)	72.3 ± 0.98	101 ± 0.45 ****	91.89 ± 0.19 *	82.59 ± 0.93 **	79.91 ± 0.93 ***

**Note:** Group I (normal group, negative control); Group II (disease model group, positive control); Group III (Ehrlich + treated with FIS loaded with gel containing 2% (*w*/*w*) HPMC); Group IV (Ehrlich + treated with FIS-NSs loaded with gel containing 2% (*w*/*w*) HPMC); Group V (Ehrlich + FIS-NSs-loaded G/HA/CMC MNs). Data are expressed as mean ± SD (n = 10). * *p* < 0.05, ** *p* < 0.01, *** *p* < 0.001, **** *p* < 0.0001, and ns (not significant) versus the control group.

## Data Availability

The raw data supporting the conclusions of this article will be made available by the authors on request.

## Abbreviations

FIS	Fisetin
NSs	*Nigella sativa* Nanovesicles
FIS-NSs	Fisetin-loaded *Nigella sativa* Nanovesicles
MNs	Microneedles
FIS-NSs-MNs	Fisetin-loaded *Nigella sativa* Nanovesicle Microneedles
GC	Gas Chromatography Analysis
HA	Hyaluronic Acid
CMC	Carboxymethyl Cellulose
SDC	Sodium Deoxycholate
TEM	Transmission Electron Microscopy
FTIR	Fourier Transform Infrared Spectroscopy
DSC	Differential Scanning Calorimetry
PDI	Polydispersity Index
IC_50_	Half Maximal Inhibitory Concentration
MMT	3-(4,5-Dimethylthiazol-2-yl)-2,5-diphenyl Tetrazolium Bromide Assay
MDA	Malondialdehyde
CAT	Catalase
SOD	Superoxide Dismutase
GSH	Reduced Glutathione
TNF-α	Tumor Necrosis Factor-Alpha
IL-1β	Interleukin-1 Beta
NF-κB	Nuclear Factor Kappa B
mTOR	Mechanistic Target of Rapamycin
BNP	Benoxinate Hydrochloride
